# A high-confidence inventory of the proteins in the mitochondrion-related organelle of *Giardia*

**DOI:** 10.1016/j.cub.2026.07.074

**Published:** 2026-10-01

**Authors:** Manish Goyal, Tiana M. Issa, Jonathan A. Stefely, Kailash Venkatraman, Jason G. McCoy, Stephanie S. Lam, Michael Z. Chen, Khanh Nguyen, Cameron Genxuan Lian, Pierre M. Jean-Beltran, Michelle Y. Fry, Bridget E. Luce, MitoCarta Tree of Life Consortium, Luke H. Chao, Steven A. Carr, Namrata D. Udeshi, Sarah E. Calvo, Vamsi K. Mootha, John Samuelson

**Affiliations:** 1 Department of Molecular and Cell Biology, Boston University, Goldman School of Dental Medicine, Boston, MA 02118, USA; 2 Department of Molecular Biology, Massachusetts General Hospital, Boston, MA 02114, USA; 3 Harvard Medical School, Boston, MA 02115, USA; 4 Broad Institute of MIT and Harvard, Cambridge, MA 02142, USA; 5 Howard Hughes Medical Institute, Massachusetts General Hospital, Boston, MA 02114, USA

**Keywords:** *Giardia*, protozoan, parasite, Metamonada, mitochondrion-related organelle, proteome, iron-sulfur cluster assembly, translocases of inner and outer membrane, ER-MRO tether, iron depletion, O_2_ exposure, anaerobic, Metronidazole, MOMTiP-4, SLC25A39, *MTM1*

## Abstract

*Giardia lamblia* is a microaerophilic protozoan parasite that infects nearly 280 million people worldwide each year. *Giardia* lacks a mitochondrial genome and mitochondrial enzymes for oxidative phosphorylation. However, it retains mitochondrion-related organelles (MROs) that house an iron-sulfur cluster (ISC) assembly pathway. To date, 32 MRO proteins have been identified with high confidence. Purifying MROs is extremely challenging due to their small size and low abundance. Here, as part of the MitoCarta Tree of Life Consortium, we used large-scale culture, density gradient fractionation, affinity purification, data-independent acquisition (DIA) mass spectrometry, protein correlation profiling, and confocal microscopy to generate the *Giardia* MitoCarta inventory (GlMitoCarta). GlMitoCarta consists of 59 proteins, 27 of which are new and 34 with unknown function. Metronidazole unexpectedly upregulates many components of the MRO. IP-MS analysis reveals that MROX6 and MROX13, proteins of unknown function, reciprocally interact and share an overlapping interactome related to ISC biology. Further, we identify MOMTiP-4 as a likely MRO inner membrane solute transporter that structurally resembles and functionally complements the yeast SLC25A carrier, Mtm1. The GlMitoCarta resource is freely available and should serve as a valuable foundation for systematic and mechanistic studies of MRO biology and *Giardia* pathogenesis.

## In Brief

Goyal *et al.* develop an optimized serial density gradient and affinity purification method to isolate tiny mitochondrion-related organelles (MROs) from the intestinal pathogen *Giardia lamblia*, enabling deep proteomic profiling and creating a new “*Giardia* MitoCarta” inventory for uncovering MRO biology and identifying potential drug targets.

## Graphical Abstract

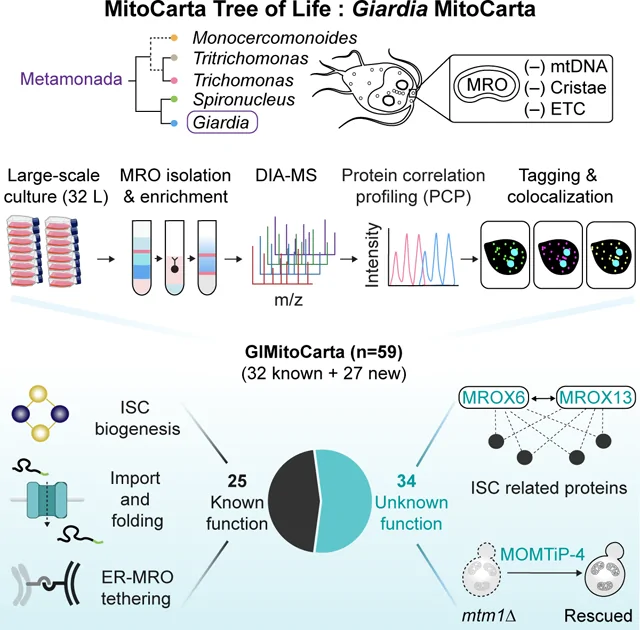

## INTRODUCTION

Mitochondrial evolution is a dynamic process driven by both adaptive and reductive changes in the organelle’s proteome. In aerobic organisms, mitochondria have maintained large macromolecular complexes to support the expression of the mtDNA genome and ATP production by oxidative phosphorylation, whereas in anaerobic or microaerophilic organisms, they have undergone reductive evolution, in some instances altogether losing genomes and aerobic pathways and adopting distinct morphological features^1–3^. A prime example is found in Metamonada, a lineage of microaerophilic and anaerobic protozoans where conventional mitochondria are either transformed into highly reduced mitochondrion-related organelles (MROs) such as mitosomes (*Giardia*) or hydrogenosomes (*Trichomonas*), or lost completely (*Monocercomonoides)*^1–5^ (Figure 1A). The only pathway common to all mitochondria and MROs is the iron-sulfur cluster (ISC) biosynthetic machinery^3,4,6^. While the mitochondrial proteomes of mammals^7,8^ and the yeast *Saccharomyces*^9^ are well-characterized, knowledge of MRO proteomes remains limited.

In this study, as part of our MitoCarta Tree of Life Consortium (MitoTOL), we focus on the MRO of the microaerophilic protist *Giardia lamblia* (also known as *Giardia intestinalis*), a flagellated intestinal parasite that causes widespread diarrheal disease in humans and animals^10,11^. *Giardia* infection is the most prevalent enteric protozoal infection, affecting nearly 280 million people worldwide each year, with approximately 1.2 million cases occurring in the United States alone^10,12^. *Giardia* trophozoites have two symmetric nuclei, which together contain a tetraploid genome that encodes approximately 5,000 proteins^10–12^. Each trophozoite contains 20–50 small (50–200 nm diameter), spherical to ovoid MROs distributed in two groups: a central array between the two nuclei and peripheral MROs scattered in the cytoplasm. Both MRO populations are associated with the endoplasmic reticulum (ER), suggesting a close relationship between MROs and the ER network^13–16^.

It has been two decades since the first report that *Giardia* possesses functional MROs^13^. *Giardia* MROs lack typical mitochondrial features (*i.e.*, DNA, cristae, and oxidative phosphorylation), while their only known function is the biosynthesis of iron-sulfur clusters (ISCs)^13,17,18^ (Figure 1A). ISC synthesis in *Giardia* is essential for cytosolic iron–sulfur protein assembly (CIA), and proteins involved in energy and amino acid metabolism, DNA replication, translation, and redox processes^17,18^. Prior investigations of the *Giardia* MRO proteome have relied on comparative genomics (homology and signal motifs)^13,19–21^, isolation of MRO-enriched subcellular fractions^22^, and affinity purification methods using tagged proteins, including enzymatic tagging and cross-linking^17,23,24^. The extreme divergence of MRO proteins, along with limited resolution and insufficient organelle purity, severely restricted these approaches.

To overcome these challenges, we combined multiple strategies, including serial density gradient fractionation, mitochondrial immunoprecipitation (MitoIP), data-independent acquisition mass spectrometry (DIA-MS)^25^, and protein correlation profiling (PCP)^26^, followed by microscopy validation to deepen the characterization of the *Giardia lamblia* MRO proteome, yielding the GlMitoCarta inventory. The GlMitoCarta consists of 59 proteins, 27 of which are new. Excitingly, 34 of these proteins are of unknown function. We demonstrate that the MRO protein MOMTiP-4 functionally complements the yeast solute carrier Mtm1. The GlMitoCarta is freely available and should serve as a resource for mechanistic and systematic studies of the MRO.

## RESULTS

### Combined density centrifugation and affinity purification yield highly enriched *Giardia* MROs

To accurately define the MRO proteome in *Giardia*, we empirically tested a variety of approaches and ultimately arrived at a protocol in which we (i) isolate MROs through a combination of differential centrifugation, density gradient centrifugation, and affinity purification; (ii) perform sensitive and quantitative proteomics analysis of successively enriched fractions by DIA-MS, and (iii) correlate the resulting profiles to three universal mitochondrial proteins using protein correlation profiling (PCP).

Of note, we began with 10 to 22 liters of *Giardia* trophozoite cultures, as the isolation protocols necessitated starting with exceptionally large biomass to obtain sufficient material for downstream analyses. To facilitate organelle isolation, we engineered parasites to express a cytosol-facing MRO protein as an affinity handle^27^. Using an episomal construct, we placed a C-terminal 3xHA-tag on the *Giardia* MRO outer membrane protein 35 (MOMP35-3xHA)^23^ (Figure 1B and Figures S1A and S1B). The tagged protein localized to both central and peripheral MROs as indicated by colocalization with the MRO marker protein Tom40^19^ (Figure 1C). Affinity purification using anti-HA magnetic beads yielded a visible bead-bound MRO pellet in MOMP35-3xHA expressing line, but not in cytosolic HaloTag-3xHA control (Figure S1C).

We developed two workflows to enrich MROs from *Giardia*. In Workflow 1, we prepared crude MROs from wild-type parasites by differential centrifugation, treated them with or without proteinase K (± PK) to remove contaminants, and then fractionated them on an optimized 10–30% OptiPrep continuous density gradient (Figure 1D). In Workflow 2, we used MOMP35-3xHA expressing parasites, prepared crude MROs by differential centrifugation, and enriched them by using an OptiPrep step gradient. We then collected the MRO-enriched layer (15–18% OptiPrep interface), subjected it to MitoIP, and further fractionated it by continuous density gradient centrifugation. To reduce organelle contamination, we treated lysates with or without digitonin (± digitonin), a non-ionic detergent that selectively disrupts membrane compartments with distinct lipid compositions (Figure 1E).

Western blot analysis using anti-GiOR-1 and anti-Tom40 (MRO markers) (Figure S1D and E), along with anti-α-tubulin (cytosol/cytoskeleton contamination) antibodies, confirms MRO enrichment in different fractions (Figure S2). In Workflow 1, we detect MROs predominantly in fractions F2–F4, with proteinase K moderately improving purity by reducing tubulin contamination (Figure S2A). In comparison, Workflow 2 enhances MRO enrichment several-fold first through step gradient centrifugation, which concentrates MROs at the 15–18% OptiPrep interface and later via affinity purification (MitoIP). Subsequent continuous gradient fractionation (10–30% OptiPrep) on the MitoIP eluate further resolves the concentrated MROs into three bands (B1–B3), with B2 and B3 showing the highest MRO enrichment compared to B1 (Figure S2B). Digitonin facilitates MRO release but also increases tubulin contamination and thus limits its overall benefit. Transmission electron microscopy (TEM) shows progressive enrichment of MROs in different fractions from Workflow 2, with band B3 appearing as the most enriched (Figure 1F). High magnification TEM micrographs show well-defined MRO compartmentalization, and measurement analysis confirms organelle integrity throughout purification (Figures S2C and S2D).

### Mass spectrometry-based protein correlation profiling to identify MRO proteins

Following quality control and validation of the MRO isolation procedures, selected fractions from Workflow 1 (± PK, n = 14) and Workflow 2 (± digitonin, n = 14) were separately subjected to mass spectrometry (MS)-based proteomic analysis, encompassing 28 samples in total (Figure 2A and Data S1). Notably, all fractions from both workflows achieved substantial protein coverage, with over 4,000 out of the total ~5,000 *Giardia* proteins identified per sample (Data S1).

To evaluate the enrichments of MROs, we created a benchmark set of 32 known MRO proteins (gold+) with strong literature confirming MRO localization (see Methods). MS analysis shows strong enrichment of benchmark gold+ proteins in both workflows (Figure 2A and Data S1). We find that gold+ proteins constitute only a small fraction of the *Giardia* total cellular proteome (~0.2%), consistent with the highly reduced MRO compared to other organisms, where mitochondria constitute 5 to 20% of the cellular proteome^7–9^. Fractionation of crude MRO from wild-type parasites results in enrichment of gold+ proteins in fractions F2–F4 relative to the whole cell and crude MRO input (Workflow 1, Figure 1D). However, the greatest absolute enrichment is achieved using a combined approach of serial desnity gradient fractionation and MitoIP (Workflow 2, Figure 1E). This approach effectively enriches gold+ proteins by ~30-fold in the affinity-purified MitoIP eluate and up to 87-fold in the continuous gradient band B3 relative to whole cell (Figure 2A). Treatment with proteinase K and digitonin further improves sample purity but yields proteomic profiles that are highly comparable to untreated samples, effectively serving as technical replicates.

Subcellular distribution during purification is further assessed using our literature-supported gold+ MRO proteins alongside annotations of subcellular compartments from GiardiaDB Gene Ontology. Heatmap analysis of 4,480 quantified proteins shows that both Workflow 1 and Workflow 2 can easily distinguish profiles from MRO versus cilium, cytoskeleton, nucleus, and cytosol, whereas Workflow 1 is better at distinguishing MRO versus extracellular region and ER proteins (Figure 2B).

While we can greatly enrich the MRO for the first time, we need to use a statistical method, protein correlation profiling (PCP)^26^, to accurately define the MRO proteome. PCP leverages many samples and replicates through which correlations with marker proteins can be more sharply and reliably ascertained. For improved sensitivity and robustness in PCP analysis, we combine samples from both Workflow 1 and Workflow 2 (n = 28) (Figure 2A) and employ three conserved mitochondrial marker proteins (Tom40, IscU, and Hspd1, also known as Hsp60). These proteins, which were selected for all the MitoTOL organisms because of their consistent expression, high conservation, and reliable mitochondrial localization, were used to identify and quantify proteins that correlate across the different purification fractions via Pearson correlation^26^ (Figure 2C and Data S2).

Based on the PCP analysis, a Pearson correlation score threshold of ≥0.95 was determined to be the most accurate for defining the mass spectrometry derived protein inventory (MitoViaPCP), identifying a total of 38 MRO proteins with a sensitivity of 81% and a false discovery rate (FDR) of 0% (100% precision) (Figures 2D and 2E and Data S2). We acknowledge that this very low FDR is driven both by our decision to select a high stringency PCP score, as well as the fact that our list of gold− proteins may be limited. A detailed examination of this dataset reveals the inclusion of 26 previously characterized gold+ proteins, along with 12 novel MRO proteins. When using a more relaxed PCP threshold of ≥0.84, 72 proteins are identified with an estimated 87% sensitivity and 37% FDR, comprising 28 gold+ proteins, and 44 new proteins (five gold−, and 39 novel MRO candidates) (Figure 2D).

### Epitope tagging and microscopy analysis

While PCP using a strict threshold helps to ensure high specificity, it misses subthreshold hits including those that may be dual-localized. Therefore, we performed extensive tagging and microscopy on 42 novel proteins with high or borderline PCP score, along with an additional 19 gold+ proteins (Figure 3A).

Of the 42 novel proteins, we initially selected all 12 newly identified MRO proteins based on PCP score ≥0.95. These proteins were C-terminally tagged with a 3xHA epitope and colocalized with *Giardia* MRO marker protein Tom40 (using anti-Tom40 antibody) as a co-stain to confirm MRO localization, which is otherwise challenging. We classified a protein as MRO only when its signals are present in both central and peripheral MROs. Reassuringly, all 12 proteins display distinct MRO localization patterns, including two (MROX16 and MROX17) that exhibit dual localization to the MRO and other subcellular structures (Figures 3B and S3A). Next, we tagged 30 sub-threshold hits (PCP scores 0.95 to 0.84) and found that five localize exclusively to the MRO, 10 localize to both MRO and other subcellular structures, and 14 show non-MRO localization (Figures S3A and S3B). One candidate (GL50803_008987) proved elusive, as transgenic parasites expressing this protein exhibit growth defects and do not survive long. Overall, we identified 27 novel MRO-localized proteins, of which 15 localize exclusively to the MRO and 12 are dual-localized to the MRO and other subcellular structures.

In addition to the above 42 proteins, we also epitope-tagged 19 gold+ proteins, including 13 that score above the PCP threshold (≥0.95) and six that score below it (0.93–0.11), to explore why gold+ proteins show variability in our PCP analysis despite their established MRO localization. Notably, all 13 high-scoring proteins and one low-scoring gold+ protein (predicted as Mitochondrial Carrier Protein (MCP))^28^ localize exclusively to the MROs. The remaining five low-scoring gold+ proteins (MOMTiP-6, MOMTiP-7, MROX3, MROX4, and MROX5) show dual localization to the MRO and other subcellular structures (Figure S4), consistent with previous reports for MOMTiP-6, MOMTiP-7, and MROX4^24,29^.

These results demonstrate the robustness of the PCP methodology for purely MRO localized proteins and underscoring the need for complementary validation using microscopy for dual localized proteins. It is worth mentioning that microscopy analysis using Tom40 colocalization clearly distinguishes the true MRO localization from MRO-like patterns (non-MRO), revealing that a disc-associated protein previously thought to be MRO instead localizes adjacent to the MRO^30^ (Figure S4).

### Defining the GlMitoCarta inventory

We define the GlMitoCarta inventory (n = 59 proteins, Figure 3C and Data S3) as the union of three sets: (i) MitoViaPCP proteins with a PCP threshold ≥0.95 (n = 38), (ii) sub-threshold hits (PCP score ≥0.84) that were experimentally validated by immunofluorescence microscopy (n = 15), and (iii) previously reported known and validated MRO proteins that scored low in our PCP analysis (n = 6). In total, the 59 GlMitoCarta proteins comprise 32 gold+ and 27 novel MRO proteins. The GlMitoCarta resource is publicly available at http://mitocarta.org.

As a collection, GlMitoCarta proteins exhibit distinctive properties when compared to the broader *Giardia* proteome (Figure S5). In general, GlMitoCarta proteins show the same distribution of abundance as the rest of the proteome, but they tend to be shorter. They also exhibit higher isoelectric points than non-MRO proteins, even after removal of the first 60 amino acids, which often contain information on mitochondrial targeting. Only eight of the 59 identified GlMitoCarta proteins possess a predicted mitochondrial targeting peptide (mTP)^31^, consistent with prior reports that protein import in *Giardia* relies on a combination of signal peptide-dependent and -independent pathways^32,33^.

For GlMitoCarta proteins lacking functional annotation, we assigned gene symbols MROX1 to MROX5 (previously known gold+ MRO genes) and MROX6 to MROX32 (27 novel MRO genes). To annotate their function, we applied computational tools to detect structural similarity (AlphaFold and Foldseek)^34,35^, remote homology (pLM-BLAST and HHpred)^36,37^, and metal binding domains^38^ (MIB2) and provide these annotations in Data S3. We could identify closely spaced cysteine residues that represent putative iron or iron-sulfur cluster (ISC) binding sites in four MRO proteins (MOMTiP-1, MROX4, MROX5, MROX12). MROX4 and MROX5 harbor both closely spaced cysteines and a LYR motif, a classic signature of ISC recipient proteins^39^.

### Comparative analysis of the *Giardia* MRO proteome

We next performed a comparative sequence analysis of the GlMitoCarta inventory. We note that such analysis is particularly challenging for *Giardia*, as it has undergone massive gene loss and secondary reduction, and many of its protein sequences have diverged into the “twilight zone” or “midnight zone” where homology detection is challenging^40^. Hence, “clade-specific” proteins likely represent a mixture of proteins that are truly clade-specific or simply diverged to the point of no longer being detectable by sequence or structural similarity.

With this caveat in mind, only 15 of the 59 GlMitoCarta proteins have detectable orthologs in the well-characterized mitochondrial proteome of human^8^ and yeast^9^. Five GlMitoCarta proteins harbor non-mitochondrial orthologs in humans and yeast, while 37 are unique to Metamonada (Figure 4A and Data S4). Compared to the mitoproteomes of humans and yeast, the *Giardia* MRO exhibits substantially more clade-specific proteins (>60%) (Figure 4B, and Data S4).

Next, we analyzed orthogroups across 203 eukaryotic species and 34 prokaryotic clades from the MitoCarta Tree of Life (MitoTOL) comparative analysis (see Chen *et al*.,^41^). Among the 59 GlMitoCarta orthologous groups, only 27% share detectable homology with prokaryotic proteins, and 12% exhibit homology across multiple eukaryotic clades (Figure 4C). The remaining 61% lack detectable homologs outside of Metamonada.

All known Metamonada species contain highly reduced MROs lacking DNA, and some lineages have lost them entirely. Even within Metamonada (Figure 4D), 35 GlMitoCarta proteins are unique to the genus *Giardia*, including 21 exclusively present in *G. lamblia* and absent in *G. muris*. The differences between *G. lamblia* and *G. muris* likely reflect evolutionary responses to their specialized environments in human and mouse hosts.

Our orthogroup analysis (Figure 4D) suggests that Oxymonadida (*e.g., Monocercomonoides exilis, Blattamonas nauphoetae, Streblomastix strix*) completely lack MROs, while Barthelonids (*e.g., Skoliomonas sp., Barthelona sp.*) are nearing complete loss, in line with observations from the literature^3^. Barthelonids have homologs to roughly 11/59 *Giardia* MRO proteins, while oxymonads have homologs to only 8/59 *Giardia* MRO proteins (Figure 4D): IscS, Hsp70, MOMTiP-7, MROX3, MROX13, MROX22, MROX27, MROX31. Notably, none of the 8 homologs identified in oxymonads were predicted to be MRO-localized (See Chen *et al*.,^41^), consistent with the complete loss of the MRO in these organisms^42^. For instance, the distantly related IscS homolog in oxymonads is a cytosolic, multidomain SufDSU fusion protein (SufD–SufS–SufU) enabling Fe–S cluster biogenesis without the mitochondrial ISC pathway^43^. Similarly, Hsp70 homologs in oxymonads appear to have a role in cytosolic protein homeostasis, rather than in the mitochondrial ISC pathway.

### IP-MS profiling of selected GlMitoCarta proteins

To better understand the functional relationship amongst MRO proteins, we performed IP-MS of six proteins (Figure S6A), including three novel (MROX6, MROX13, MROX14) and three previously known MRO members: MOMP35^23^ (outer membrane protein of unknown function), MOMTiP-1 (now predicted to bind ISC in our computational analysis), and MOMTiP-4^24^ (now predicted to resemble SLC25A transporters).

Because the MRO content of *Giardia* is so tiny (~0.2%), it is preferable to use MRO-enriched crude preparations for immunoprecipitation rather than whole-cell lysates. Although prior *Giardia* IP studies additionally relied on chemical crosslinking^17,24^, which increases sensitivity, we chose to proceed without these agents as they can introduce false positives. We performed each IP in biological duplicate across three independent batches and paired each batch with corresponding replicates of WT MRO lysate IP as a control (Figure S6A). Across all IP-MS experiments, we identified 2,275 proteins, including all gold+ proteins (Data S5).

IP-MS clearly validated both novel and previously known MRO proteins, as GlMitoCarta proteins (gold+ and novel) were strongly enriched in pulldowns from all six baits and the baits themselves consistently scored as the top hits (Figure S6B and Data S5). MROX6 and MROX13 are notable as they pulled each other down as high-scoring hits (>10x enrichment, adj. p-value <1e–5) as well as a highly overlapping GlMitoCarta interactome consisting of: BolA, Tim44, RhodaneseLP, IscU, Fdx, Hsp70, Glrx5, MROX1, MROX2, MROX10 (Figure S6C). These IP-MS data represent a valuable resource for future in-depth studies and indicate that MROX6 and MROX13 are functionally related to each other as new components of the ISC pathway.

### The *Giardia* MRO is responsive to environmental and drug perturbations

Next, we sought to determine to what extent the GlMitoCarta proteome remodels in response to key drug and environmental perturbations. We subjected *Giardia* trophozoites to metronidazole (MTZ), the first-line therapy for giardiasis^44,57^ and performed both transcriptional and proteomic profiling. We also subjected the pathogen to iron deprivation and O_2_ exposure and profiled the resulting transcriptional responses.

MTZ treatment results in stunted growth, reduced motility, and impaired attachment, alongside significant morphological aberrations, including parasite clumping and the formation of large cytoplasmic vesicles. Our RNA-seq data indicate no concordant changes in the MRO proteome at the RNA level (Figure 5A and Data S6). However, mass spectrometry reveals remodeling of the GlMitoCarta proteome, with 6 of 51 detected GlMitoCarta proteins significantly changing with MTZ (p-value <0.01 based on t-test) (Figure 5B and Data S7). In fact, Gene Set Enrichment Analysis (GSEA) shows significant upregulation of GlMitoCarta proteins with MTZ treatment (q-value <1e-4), but no significant change from any other cellular compartment (Figure 5C). Notably, MTZ upregulates key ISC pathway proteins (IscS, Nfu1), MOMTiP-2, MPP, Cpn60, as well as the novel MRO protein MROX7 (Figure 5D and Data S7). These results highlight the potential role of MRO proteins, especially those in the ISC pathway, in response to MTZ treatment.

Iron depletion and O_2_ exposure also reduced parasite growth and altered morphology, along with changes in the transcript levels of several GlMitoCarta genes. For O_2_ exposure, the peak changes were observed at 8 hours compared to the 0-hour controls, while recovery under anaerobic conditions (De-ox 4h) restored gene expression to baseline (Figure 5E and Data S6). The most notable changes were in key ISC pathway genes (IscS, IscU, Fdx), Tom40, MOMTiP-2, and four novel MRO genes (MROX10, MROX14, MROX20, and MROX28), which showed significant upregulation (DESeq2 adjusted p-value <0.01) under both iron depletion (−Iron/+Iron) and O_2_ exposure (8 h O_2_ /0 h). In contrast, MOMP35, MROX4, and MROX11 are downregulated in both these conditions (Figure 5E). Collectively, these findings suggest a coordinated transcriptional response to iron depletion and O_2_ exposure, involving ISC and GlMitoCarta genes of unknown function.

### MOMTiP-4 functionally complements yeast Mtm1

Based on our computational analyses (Data S3), we were particularly interested in MOMTiP-4, which has previously been reported to be localized to the MRO^23,24^, and which we validated via PCP analysis (PCP score ≥0.95) and microscopy (Figure S4). Remote homology searches performed using pLM-BLAST showed that MOMTiP-4 exhibits weak similarity at its N-terminus to the SLC25A family of mitochondrial transporters. The AlphaFold 3.0^45^ predicted structure of MOMTiP-4 contains six amphipathic helices aligned to form a pore resembling other SLC25A family members (Figure 6A). Although the IP-MS analysis did not provide detailed insight about its function (Figure S6), these computational analyses raise the provocative hypothesis that MOMTiP-4 may be a solute transporter across the MRO inner membrane.

To investigate this possibility, we performed functional complementation in yeast *S. cerevisiae*. We found that MOMTiP-4 could be heterologously expressed in yeast and detected in the mitochondrial fraction (Figure 6B). We then expressed MOMTiP-4 in the background of five single knockouts (*aac2*Δ, *leu5*Δ, *mir1*Δ, *mtm1*Δ, *sam5*Δ) and two double knockouts (*ndt1*Δ*ndt2*Δ*, mrs3*Δ*mrs4*Δ*)* of yeast SLC25A family members, which exhibit growth defects that are more pronounced on non-fermentable substrates. These yeast SLC25A mitochondrial carrier family members are involved in the transport of key metabolites and ions, including ADP/ATP (*AAC2*), coenzyme A (*LEU5*), phosphate (*MIR1*), metal ions (*MTM1*), S-adenosylmethionine (*SAM5*), NAD^+^ (*NDT1* and *NDT2*), and iron (*MRS3* and *MRS4*).

In these genetic complementation analyses, we found that MOMTiP-4 partially rescues the growth defect in *mtm1*Δ cells but does not affect the other tested strains (Figures 6C and S7). The rescue is observed on a non-fermentable substrate when mitochondrial function is required. Yeast Mtm1 has previously been reported to serve as a pyridoxal-5’-phosphate (PLP) transporter^46^ and is evidently critical for iron homeostasis^47^, while its human homolog SLC25A39 in recent years has been reported to serve as a glutathione transporter ^48,49^. To test whether MOMTiP-4 complements the iron accumulation phenotype in *mtm1*Δ cells, we compared the iron content of isolated mitochondria from wild type, *mtm1*Δ, and *mtm1*Δ cells expressing MOMTiP-4. Interestingly, we found that MOMTiP-4 expression prevents mitochondrial iron accumulation in the *mtm1*Δ background (Figure 6D), implying a role of MOMTiP-4 in iron homeostasis. Our studies indicate that MOMTiP-4 may serve as a solute transporter involved in maintaining iron homeostasis within the *Giardia* MRO. To the best of our knowledge, this is the first time that a *Giardia* protein has functionally complemented a knockout of a yeast inner membrane solute transporter.

### GlMitoCarta proteins and pathways

The small size of *Giardia* MitoCarta (n = 59) makes it feasible to comprehensively enumerate all functional pathways (Figure 7): (i) ISC assembly (n = 9), (ii) protein import, processing, and folding (n = 11), and (iii) ER-MRO tethers involved in lipid exchange and mitochondrial dynamics (n = 5). The remaining category is (iv) proteins of unknown function (n = 34).

Using our dataset and previous literature studies^17,18^, we classify a total of nine proteins as being related to iron sulfur cluster biogenesis. We include eight ISC assembly proteins in *Giardia*, including Glrx5, IscA, Fdx, Nfu1, BolA, IscU, IscS, and RhodaneseLP. We also place in this category GiOR-1, an MRO-localized NADPH-dependent diflavin oxidoreductase^23,50^ which has previously been reported to interact in a robust manner with ISC machinery^17^. It is possible that GiOR-1 may help with NADPH-dependent electron transfer in the ISC pathway, though this hypothesis requires further experimental validation.

We catalog eleven proteins associated with MRO protein import, folding, and processing. This group includes chaperonins (Cpn10 and Cpn60), heat shock protein Hsp70, co-chaperones (Jac1, also known as DnaJ), elements of the pre-sequence translocase-associated motor (PAM) complex (Pam16, Pam18), and translocase subunits (Tom40, Tim17, Tim44). Additionally, nucleotide exchange factors (Mge1) and a MPP (mitochondrial processing peptidase) are also categorized in this pathway. Notably, Jac1, Hsp70, and Mge1 also crosstalk and play key roles in ISC assembly by functioning as specialized chaperones^17^ (Figure 7).

We catalog a total of five proteins associated with ER-MRO tethering. Although previous studies suggested that *Giardia* lacks the canonical ER mitochondria encounter structure (ERMES)^51,52^, a recent study identified related proteins^24^. These proteins localize either strictly to the MROs (MOMTiP-1, MOMTiP-2, MOMTiP-3/MOMP35, and MOMTiP-4) or to both the MROs and the ER (MOMTiP-5, MOMTiP-6, MOMTiP-7, and MOMTiP-8). Interestingly, MOMTiP-7 and MOMTiP-8 share sequence similarity with SNARE proteins and yeast Mmm1, respectively^24^. Our independent GlMitoCarta analysis confirms the localization of MOMTiP-1 to MOMTiP-4 exclusively to MROs, and MOMTiP-6 and MOMTiP-7 to both ER and MRO, supporting their possible role in forming ER-MRO tethers as in other organisms (Figure 7). In our study, we find that MOMTiP-4 functionally complements yeast Mtm1, a SLC25A family transporter, and therefore have not grouped it in the ER–MRO tethering pathway.

Excitingly, our dataset contains a total of 34 proteins of unknown function that may represent *Giardia*-specific innovations. Our preliminary studies indicate that MROX6 and MROX13 may be interacting either directly or indirectly together with the broader ISC production machinery. We also provide strong functional evidence that MOMTiP-4 is a solute transporter through our genetic complementation study. However, the majority of MRO proteins await functional characterization (Figure 7 and Data S3).

## DISCUSSION

Until two decades ago, it was believed that *Giardia* do not contain mitochondria. The landmark discovery of mitochondrion-related organelles (MRO)^13^ containing Fe–S clusters was pivotal in revising our textbook view of eukaryotic evolution and underscoring the importance of reductive evolution in shaping extant mitochondria. Since then, efforts to characterize the *Giardia* MRO have been challenging both from an experimental perspective (because of the small size relative to broader cellular mass) and their extreme evolutionary divergence (hampering sequence-based analysis). Here, as a part of the MitoTOL consortium, we have used a combination of methods to accurately characterize the *Giardia* MRO proteome. The GlMitoCarta, along with other MitoTOL Consortium MitoCarta datasets, is available at http://mitocarta.org.

A comparison of the *Giardia* MRO proteome with those of other anaerobic protists reveals that it is exceptionally streamlined and lacks specific metabolic evolutions such as the sulfate activation pathway present in the *Entamoeba*^53^, the alternative electron transport chain in *Cryptosporidium*^54^, or glycine cleavage system in *Trimastix*^55^. Likewise, in the endo-parasitic protist *Paramikrocytos*, the MRO is predicted to participate in ATP generation and de novo phospholipid biosynthesis^56^. Instead, the MRO in *Giardia* is enriched in ISC assembly components and associated chaperones, emphasizing its central role in Fe–S cluster biogenesis either to feed the cytosolic iron-sulfur protein assembly (CIA) pathway or to support the maturation of MRO-localized ISC-containing proteins. Our perturbation profiling identifies the ISC pathway as responsive to metronidazole treatment. Metronidazole is a prodrug that, upon reductive activation, generates reactive intermediates which can indirectly affect Fe–S cluster dependent and other redox-sensitive proteins^44,57^. Comparative studies of metronidazole-resistant strains have also reported broad remodeling of ISC-related components, reflecting adaptive responses to drug toxicity^58–60^. Likewise, the upregulation of ISC proteins under iron depletion may reflect a compensatory and/or coordinated adaptive response, consistent with observations across other eukaryotic systems where iron starvation affects ISC genes^61,62^.

In our study, MOMTiP-4 was of outstanding interest, and to the best of our knowledge, it is the first *Giardia* protein to functionally complement a missing SLC25A transporter in yeast. A recent study used iterative sequence profile searches to spotlight a *Giardia* protein as a homolog of the human mitochondrial solute carrier SLC25A43 and classified it as a mitochondrial carrier protein (MCP). Although they could demonstrate the protein localization to the MRO, its function as a solute carrier remains to be validated due to challenges in heterologous expression^28^. Here, MOMTiP-4 emerged as a strong candidate transporter in *Giardia*, predicted to adopt a canonical mitochondrial carrier fold composed of six pseudo-symmetrical amphipathic helices characteristic of the SLC25A family. MOMTiP-4 partially rescued respiratory growth in yeast cells lacking Mtm1, a putative PLP transporter^46^ and reversed the mitochondrial iron accumulation phenotype that is characteristic of *mtm1*Δ cells^63^ (Figure 6). PLP is an essential cofactor for proteins that facilitate iron-sulfur cluster biogenesis (such as IscS),^64,65^ and indeed, *mtm1* knockout strains exhibit pleiotropic phenotypes associated with iron dysregulation including: mitochondrial iron overload^63^, reduced activities of superoxide dismutase 2 ^47^ and Fe–S cluster-containing enzymes^66^. Additionally, *mtm1* mutants show synthetic lethal interactions with glutathione synthesis genes (*gsh1* and *gsh2*)^49^ suggesting a parallel role in redox homeostasis. This is further supported by the observation that a mammalian homologue of Mtm1, the glutathione transporter SLC25A39 rescues respiratory growth defects in yeast *mtm1*Δ cells^67^. Collectively, these observations indicate that MOMTiP-4 serves as an important MRO solute transporter, potentially mediating the transport of small metabolites PLP (required for IscS activity) or glutathione (a redox cofactor), with direct or secondary impact such as iron transport and homeostasis (ISC biogenesis). Future work will therefore need to define its substrate and role in the MRO.

An exciting future challenge lies in deciphering the roles of the remaining GlMitoCarta proteins of unknown function (Figure 7). An open question is whether these proteins participate in the three established pathways (protein import, ISC biogenesis, ER-MRO tethering) or instead participate in other pathways. We anticipate that uncovering their function will require a combination of modern computational and experimental approaches, as we have illustrated for MOMTiP-4. Such studies are expected to provide much deeper insights into the organelle’s evolutionary “reduction” and metabolic diversity within eukaryotes.

### Limitations of the study

We isolated MROs under standard laboratory conditions while briefly exposing them to atmospheric oxygen (21% O_2_). *Giardia* is a microaerophilic organism, and O_2_ exposure may compromise the MRO stability. Future studies using an anaerobic chamber to isolate MRO under strictly anaerobic conditions may enhance protein stability. This study primarily focuses on the trophozoite stage of *Giardia*, which is biochemically tractable for MRO isolation. Exploring cyst stages may reveal insights into how *Giardia* adapts its organelles to different environmental conditions and developmental cues.

A key limitation of our study is the very low expression of some MRO proteins when expressed with an episomal plasmid, which complicates their detection due to a high background signal. Additionally, some lines exhibited overexpression-related effects, including slow growth, low population with positive signals and extremely low MRO signals. This underscores the need for promoter optimization or endogenous tagging, particularly for non-MRO labelled proteins. Here, we have attempted to address this issue by using the Tom40 antibody to mark MRO structures, which greatly improved localization accuracy and enabled us to define the GlMitoCarta.

More than half of the GlMitoCarta protein inventory is of unknown function and awaits further detailed analysis. As we indicated, computational analysis remains challenging because of high sequence divergence. We identified four MRO proteins (MROX4, MROX5, MROX12 and MOMTiP-1) as potential ISC binders, which require further experimental validation (*e.g.*, ^55^Fe incorporation followed by immunoprecipitation of epitope-tagged proteins). We have been able to functionally complement yeast *MTM1* with MOMTiP-4, but future studies are required to determine the specific substrate for this transporter.

## RESOURCE AVAILABILITY

### Lead contact

Further information and requests for resources and reagents should be directed to and will be fulfilled by the lead contact, John Samuelson (jsamuels@bu.edu).

### Materials availability

*G. lamblia* strains and plasmids generated in this study are available upon request from the lead contact.

### Data and code availability

- Proteomics and transcriptomics datasets are available in the supplementary Data S1–S7.
- The original mass spectra and the protein sequence databases used for searches have been deposited in the public proteomics repository MassIVE (http://massive.ucsd.edu) and are accessible under MassIVE ID MSV000101929.
- Raw RNA-seq transcriptomic datasets have been deposited in NCBI Gene Expression Omnibus (GEO) and are accessible under GEO accession number GSE339042.
- Confocal microscopy data have been deposited in the BioImage Archive (https://www.ebi.ac.uk/bioimage-archive) under accession number S-BIAD3383.
- GlMitoCarta resource is available at http://mitocarta.org.
- Any additional information required to reanalyze the data reported in this paper is available from the lead contact upon request.

## STAR★METHODS

### EXPERIMENTAL MODEL AND STUDY PARTICIPANT DETAILS

#### *Giardia lamblia* strain maintenance

*Giardia lamblia* strain WB (ATCC 50803/WB clone C6) was routinely cultured under microaerophilic conditions. Trophozoites were maintained in modified TYI-S-33 medium, supplemented with 10% adult bovine serum and bovine bile as per the Keister method^68^. Cultures were incubated without shaking at 37 °C in sterile 12.5 cm plug-seal cap tissue culture flasks (Fisher Scientific, Cat. # FB012934). The flasks were filled up to the neck with media to maintain anaerobic conditions.

### METHOD DETAILS

#### Cloning and transfections

##### Molecular cloning of plasmids

DNA constructs were cloned into the episomal circular plasmid pKS-3xHA-PAC with minor modifications (From Alex Paredez, University of Washington), originally designed for endogenous tagging ^69^. For episomal expression of *Giardia* proteins, the Glutathione synthase gene promoter (GL50803_007195) was used unless otherwise stated. For construct preparation, the coding region of the target gene and vector backbone were PCR amplified using Q5^®^ Hotstart Master Mix 2x (New England BioLabs, Cat. #M0494S). PCR fragments were then assembled using NEBuilder HiFi DNA Assembly Master Mix (New England Biolabs, Cat. #E2621). The assembled plasmid was transformed into NEB^®^ 5-alpha Competent *E. coli* cells (New England Biolabs, Cat. #C2987H) for scale-up and preparation. Isolated plasmids were sequenced via whole plasmid sequencing.

##### Plasmid transfections

Prior to the day of transfection, *Giardia lamblia* strain WB, wild-type trophozoites, were cultured in early log phase growth (confluency 60–70%) (axenic cultures, standard media, sealed side-arm tissue culture flasks, 37 °C, stationary dry incubator). On the day of transfection, trophozoites were harvested by incubating on ice (30 min, 4 °C) to detach the adherent cells, transferring the cell suspension to a 50-mL conical tube, and pelleted the cell by centrifugation (1500 *g*, 10 min, 4 °C). The supernatant was decanted; cells were gently resuspended in chilled standard media and manually counted. In pre-chilled electroporation cuvettes (Gene Pulser/MicroPulser Electroporation Cuvettes, 0.4 cm, Cat. # 1652088), 0.3 mL of resuspended cells (1×10^7^ cells) were added along with plasmid DNA (20–50 μg in a sterile tube), gently mixed by tapping, and incubated the mixture on ice (15 min, 4 °C). Cells were then electroporated using a Gene Pulser Xcell Electroporation System (Bio-Rad Laboratories, 375 V, 1000 μF, and R10/720 Ω). After electroporation, we gently flicked the cuvettes to mix the contents, allowed them to rest for 15 minutes at 4 °C, then transferred the cells into sterile, plug-seal capped 12.5-cm side-arm culture flasks filled to the neck with pre-warmed culture medium, and incubated them for recovery for 24 hours at 37 °C. One day post transfection, the drug selection was initiated by replacing the spent media with fresh media containing the appropriate selective antibiotic for the construct: Puromycin (50 μg/mL, InvivoGen, Cat. #ant-pr-5b) or Blasticidin (70 μg/mL, InvivoGen, Cat. # ant-bl-5b). The culture media with appropriate selection was replaced every other day until trophozoites began to proliferate.

#### Isolation of MROs

##### Preparation of isotonic OptiPrep gradients

OptiPrep (Sigma, Cat. # D1556) is supplied as a 60% iso-osmotic iodixanol suspension in water. Here, this solution is considered “60% OptiPrep”. 50% OptiPrep “Iodixanol Working Solution” (IWS, 0.28 M sucrose, 10 mM HEPES, 0.2 mM EDTA, pH 7.2, 50% OptiPrep v/v) was prepared by diluting 60% OptiPrep and “OptiPrep Diluent” buffer (0.28 M sucrose, 60 mM HEPES, 1.2 mM EDTA, pH 7.2) in 5:1 ratio. Lower percentages (10%, 15%, 18%, and 30%) of isotonic OptiPrep were prepared by mixing the appropriate volumes of IWS and “1X SHE” buffers (0.28 M sucrose, 10 mM HEPES-KOH, pH 7.2, 0.2 mM EDTA) with 0.25 mM PMSF. Immediately prior to use, 10–30% continuous gradient was prepared using a gradient maker (Gradient Master IP, BioComp Instruments) in a Seton Scientific ultracentrifuge tube (14 × 89 mm, Polypropylene, Cat # 7030).

Additionally, immediately prior to use, a four-step gradient of OptiPrep was prepared on ice in Beckman Coulter ultracentrifuge tubes (16 × 102 mm, Ultra-Clear, Cat # 344061) (bottom layer, 3 mL of 30% isotonic OptiPrep; bottom middle layer, 3 mL of 18% isotonic OptiPrep; top middle layer, 3 mL of 15% isotonic OptiPrep; top layer, 3 mL of 10% isotonic OptiPrep).

##### Isolation of MROs from wild type parasite (Workflow 1, density gradient)

*Giardia lamblia* strain WB, wild type, was maintained in log phase growth in standard media for three days prior to the experiment. The experimental cultures (175-cm plug-seal flasks, 700 mL standard medium) were inoculated with 1×10^7^ trophozoites and incubated (37 °C, stationary, 72 h) until cells reached confluency. For each biological replicate, fifteen 175-cm flasks (affording approximately 1×10^10^ cells after harvesting from 10 liters of total culture volume) were used for a single preparation of MROs.

Both adherent and non-adherent cells were harvested. Adherent cells were detached by ice (30 min, 4 °C) and transferred to 500 mL wide-mouth centrifuge bottles (Beckman Coulter, Cat. # 355607). The harvested cells were centrifuged (3,000 *g*, 10 min, 4 °C) to collect intact cells. The resulting cell pellet was resuspended with 1X SHE buffer (0.28 M sucrose, 10 mM HEPES-KOH, pH 7.2, 0.2 mM EDTA) containing 0.25 mM PMSF and then centrifuged (3,000 *g*, 10 min, 4 °C) to collect washed intact cells. The supernatant was decanted and disposed of, and the cell pellet was resuspended in 1X SHEP buffer (1X SHE, 1x Roche cOmplete^™^ Protease Inhibitor Cocktail, 0.25 mM freshly added PMSF) (final volume to 160 mL, 4 °C). Following cell harvesting, all subsequent procedures in the laboratory were meticulously carried out at 4 °C, either in a cold room or on ice, to ensure optimal conditions. This included the consistent use of pre-cooled equipment and freshly prepared buffers.

Aliquots of whole-cell samples (50 μL) were centrifuged (10,000 *g*, 10 min, 4 °C) to isolate washed cell pellets, then processed as follows: (i) frozen in N_2(l)_ for western blots and proteomics and(ii) resuspended in PBS (100 μL 4 °C), mixed (1:1 v/v) with “2x FGP” fixative (2.5% paraformaldehyde, 5.0% glutaraldehyde, 0.06% picric acid in 0.2 M cacodylate buffer, pH 7.4) (100 μL, freshly prepared), and stored at 4 °C, protected from light, for electron microscopy (final fixative concentrations: 1.25% paraformaldehyde, 2.5% glutaraldehyde, 0.03% picric acid in 0.1 M cacodylate buffer, pH 7.4).

The rest of the cell suspension was transferred to a 250-mL plastic conical tube and mechanically lysed by nitrogen cavitation (Cell Disruption vessel, Parr Instruments). The system with cell suspension was allowed to equilibrate on ice (1100 PSI, 15 min, 4 °C). The content was discharged from the vessel through the release valve at a flow rate of approximately two droplets per second into another 250-mL tube on ice. The discharged material was returned to the vessel in a new 250-mL tube and again allowed to equilibrate on ice (1100 PSI, 15 min, 4 °C). The content was again discharged from the vessel through the release valve at a flow rate of approximately two droplets per second. The whole-cell homogenate was transferred into 50-mL Eppendorf tubes on ice. The combined cell homogenates were centrifuged at slow speed (10,000 *g*, 15 min, 4 °C) to pellet intact cells and nuclei. The slow-spin supernatant was recovered, transferred to a clean 50-mL Eppendorf tube on ice, and centrifuged again at slow speed (10,000 *g*, 15 min, 4 °C) to clear any remaining intact cells or nuclei. The slow-spin supernatant was recovered and transferred to a 38.5-mL Beckman Coulter ultracentrifuge tube (25 × 89 mm, Ultra-Clear, Cat. # C14292) on ice. In the cold room, 1.5 mL 60% OptiPrep was underlaid beneath the homogenate, creating a cushion to protect cell integrity during ultracentrifugation. The homogenate was then ultracentrifuged (SW 32 Ti rotor, 100,000 *g*, 30 min, 4 °C) to form a crude MRO band above the 60% OptiPrep cushion. The high-speed supernatant was aspirated and disposed. The resulting band was mixed with the OptiPrep to a final volume of 8 mL with a density of 36% OptiPrep.

5 mL aliquot of this resuspension was split (2.5 mL) and transferred to 15-mL Falcon tubes, labeled −PK and +PK. The +PK Falcon tube was treated with 1 μg/mL Proteinase K and incubated (10 min, 4 °C). The reaction was quenched with 1 mM PMSF and 50x protease inhibitor cocktail (to a final concentration of 2x protease inhibitor).The −PK Falcon tube was left untreated and on ice for 10 min. −PK and +PK were each underlaid beneath a 10–30% OptiPrep continuous gradient (see details above in separate methods section) in a Seton Scientific ultracentrifuge tube and ultracentrifuged (SW 41 Ti rotor, 120,000 *g*, 2 h, 4 °C).

The remaining crude MRO resuspension was transferred to a new Seton Scientific ultracentrifuge tube, diluted with 1X SHE (final volume to 11 mL, 4 °C) and pelleted by centrifugation (120,000 *g*, 30 min, 4 °C). The resultant pellet was gently resuspended in 1X SHE (300 μL, 4 °C) and split into aliquots (i) frozen in N_2(l)_ for western blots and proteomics or (ii) resuspended in PBS (100 μL, 4 °C), mixed with 2x FGP fixative (100 μL, freshly prepared) and stored at 4 °C, protected from light, for electron microscopy.

After ultracentrifugation, each gradient was placed in a fractionation machine (PGFip, Piston Gradient Fractionator, BioComp Instruments) and 12 fractions were collected, labeled F1–F12 for −PK and +PK gradients. F1–F9 and F11 were 0.84 mL fractions collected directly from the machine (0.2 speed, 6 mm distance). F10 was the result of combining two fractions for 1.68 mL total volume to increase low protein concentration. F12 was the material remaining at the bottom of the tube that could not be directly collected by the fractionation machine. Each fraction was transferred to a fresh Seton Scientific tube, diluted in 1X SHE (to a final volume of 11 mL, 4 °C) and centrifuged (120,000 *g*, 20 min, 4 °C). The resultant pellet was gently resuspended in 1X SHE (200 μL, 4 °C) and split into aliquots. (i) frozen in N_2(l)_ for western blots and proteomics or (ii) resuspended in PBS (100 μL, 4 °C), mixed with 2x FGP fixative (100 μL, freshly prepared) and stored at 4 °C, protected from light, for electron microscopy.

##### Isolation of MRO from MOMP35-3xHA transfected parasites (Workflow 2, MitoIP and density gradient)

For MitoIP*, Giardia lamblia* strain WB, was transfected with a plasmid pKS-Epi_Puro_GS_GL50803_0014939 (MOMP35)-3xHA encoding *Giardia* MRO Outer Membrane Protein 35 (MOMP35, GL50803_0014939), tagged at its C-terminus with 3xHA tag. The transgenic parasites were maintained in log phase growth in standard media for three days before the experiment. The experimental cultures (225-cm plug-seal flasks, ~1,000 mL standard medium) were inoculated with 1×10^7^ trophozoites and incubated (37 °C, stationary, 72 h) until cells reached confluency. For each biological replicate, twenty-two 225-cm flasks (affording approximately 1.5 × 10^10^ cells after harvesting from 22 liters of total culture volume) were used for a single preparation of MROs.

Both adherent and non-adherent cells were harvested. Adherent cells were detached by incubating on ice (30 min, 4 °C) and transferred to 500 mL wide-mouth centrifuge bottles (Beckman Coulter, Cat. # 355607). The harvested cells were centrifuged (3000 *g*, 10 min, 4 °C) to collect intact cells. The resulting cell pellet was resuspended with 1X SHE buffer (0.28 M sucrose, 10 mM HEPES-KOH, pH 7.2, 0.2 mM EDTA) containing 0.25 mM PMSF and then centrifuged (3,000 *g*, 10 min, 4 °C) to collect washed intact cells. The supernatant was decanted and disposed of, and the cell pellet was resuspended in 1X SHEP buffer (1X SHE, 1x Roche cOmplete^™^ Protease Inhibitor Cocktail, 0.25 mM freshly added PMSF) (final volume to 160 mL, 4 °C).

Aliquots of whole-cell samples (50 μL) were centrifuged (10,000 *g,* 10 min, 4 °C) to isolate washed cell pellets, then processed as follows: (i) frozen in N_2(l)_ for western blots and proteomics and (ii) resuspended in PBS (100 μL 4 °C), mixed (1:1 v/v) with “2x FGP” fixative (2.5% paraformaldehyde, 5.0% glutaraldehyde, 0.06% picric acid in 0.2 M cacodylate buffer, pH 7.4) (100 μL, freshly prepared), and stored at 4 °C, protected from light, for electron microscopy (final fixative concentrations: 1.25% paraformaldehyde, 2.5% glutaraldehyde, 0.03% picric acid in 0.1 M cacodylate buffer, pH 7.4).

The rest of the cell suspension was transferred to a 250-mL plastic conical tube and mechanically lysed by nitrogen cavitation (Cell Disruption vessel, Parr Instruments). The system with cell suspension was allowed to equilibrate on ice (1100 PSI, 15 min, 4 °C). The content was discharged from the vessel through the release valve at a flow rate of approximately two droplets per second into another 250-mL tube on ice. The discharged material was returned to the vessel in a new 250-mL tube and again allowed to equilibrate on ice (1100 PSI, 15 min, 4 °C). The content was again discharged from the vessel through the release valve at a flow rate of approximately two droplets per second. The whole-cell homogenate was transferred into 50-mL Eppendorf tubes on ice. The combined cell homogenates were centrifuged at slow speed (10,000 *g*, 15 min, 4 °C) to pellet intact cells and nuclei. The slow-spin supernatant was recovered, transferred to a clean 50-mL Eppendorf tube on ice, and centrifuged again at slow speed (10,000 *g*, 15 min, 4 °C) to clear any remaining intact cells or nuclei. The slow-spin supernatant was recovered and transferred to a 38.5-mL Beckman Coulter ultracentrifuge tube (25 × 89 mm, Ultra-Clear, Cat. # C14292) on ice. In the cold room, 1.5 mL 60% OptiPrep was underlaid beneath the homogenate, creating a cushion to protect cell integrity during ultracentrifugation. The homogenate was then ultracentrifuged (SW 32 Ti rotor, 100,000 *g*, 30 min, 4 °C) to form a crude MRO band above the 60% OptiPrep cushion. The high-speed supernatant was aspirated and disposed. The resulting band was mixed with the OptiPrep to a final volume of 8 mL with a density of 36% OptiPrep.

The resuspended crude MRO was distributed into six 17-mL Beckman Coulter ultracentrifuge tubes, and a freshly prepared four-step (30%, 18%, 15%, 10%) isotonic OptiPrep gradient (see details above in separate methods section) was layered on top. The step-gradient was subsequently ultracentrifuged (SW 32.1 Ti rotor, 120,000 *g*, 1.5 h, 4 °C).

The remaining resuspended crude MRO sample was transferred to a 17 mL Beckman ultracentrifuge tube, diluted with 1X SHE (final volume to 12 mL, 4 °C), and ultracentrifuged (120,000 *g*, 30 min, 4 °C). The resultant pellet was gently resuspended in 1X SHE (300 μL 4 °C), split into aliquots and processed as follows: (i) frozen in N_2(l)_ for western blots and proteomics and (ii) resuspended in PBS (100 μL, 4 °C), mixed with 2x FGP fixative (100 μL, freshly prepared) and stored at 4 °C, protected from light, for electron microscopy.

After ultracentrifugation, four bands were distinctly seen at the interfaces between each step of the gradient (e.g., 10–15%, 15–18%, 18–30% and 30–36% OptiPrep). From a single tube, all four bands were collected by puncturing the side of the ultracentrifuge tube with a 5-mL syringe and an 18-G needle, diluted in 1X SHE (to a final volume of 11 mL), and centrifuged (100,000 *g*, 30 min, 4 °C). The resultant pellets were gently resuspended in 1X SHE and split into aliquots, then frozen in N_2(l)_ for western blot analysis. Only the 15–18% interface had additional aliquots (i) frozen in N_2(l)_ for proteomics or (ii) resuspended in 100 μL of 1X PBS (4 °C) then combined 1:1 (v/v) with 100 μL of freshly prepared 2x FGP fixative, stored at 4 °C, and protected from light for electron microscopy (since 15–18% interface contained the greatest MRO purity). The band at the 15–18% OptiPrep interface was collected from the rest of the tubes by puncturing the side of the ultracentrifuge tube with a 5-mL syringe and an 18-G needle. The pooled extraction was then diluted in 1X SHEP (to a final volume of 50 mL, 4 °C) and incubated with 300 μL anti-HA microbeads (Miltenyi^™^ HA Isolation Kit, Miltenyi Biotec, Cat. # 130-091-122) and incubated (1 h, 4 °C, gentle rotation).

Working at 4 °C in the cold room, MACS LS columns (Miltenyi Biotec, Cat. # 130-042-401) were placed in a magnetic QuadroMACS multi-stand separator (Miltenyi Biotec, Cat. # 130-090-976) and pre-equilibrated with 4 mL of 1X SHE buffer. To eliminate cell aggregates or large particles, a 30 μm pre-separation filter (Miltenyi Biotec, Cat. # 130-041-407) was placed atop the column. The immunocapture of MROs began by passing the sample through the column in 4-mL steps. The columns were then washed four times with 1X SHE buffer (4 mL, 4 °C). Then, the columns were removed from the magnetic separator and the immunocaptured MROs were gently eluted with 1X SHE (4 mL, 4 °C) using a plunger. A small portion of the MitoIP eluate (2.0 mL) was further a aliquoted and centrifuged (18,000 *g*, 30 min, 4 °C). The supernatant was discarded, and the isolated pellets were then (i) frozen in N_2(l)_ for western blots and proteomics and (ii) resuspended in PBS (100 μL, 4 °C), mixed with 2x FGP fixative (100 μL, freshly prepared) and stored at 4 °C, protected from light, for electron microscopy.

The remaining MitoIP eluate (14 mL) was transferred to a 17-mL ultracentrifuge tube and 0.8 mL of 60% OptiPrep was carefully underlaid beneath the sample and centrifuged (100,000 *g*, 30 min, 4 °C) to form a concentrated band above the OptiPrep cushion. The high-speed supernatant was aspirated and disposed. The resulting band was mixed with the OptiPrep to a final volume of 2.5 mL with a density of 36% OptiPrep. The resuspended material was underlaid beneath a freshly prepared 10–30% OptiPrep continuous gradient (see details above in separate methods section) and centrifuged (120,000 *g*, 1 h, 4 °C). After ultracentrifugation, three bands were seen: a top band (roughly at 15% OptiPrep, B1), a middle band (roughly at 25% OptiPrep, B2), and the bottom band (at the 30–36% interface, B3). The same extraction with an 18-G needle and 5-mL syringe was repeated to extract continuous bands B2 and B3. These bands were transferred to a 17-mL ultracentrifuge tube and diluted with 1X SHE (to a final volume of 12 mL) and centrifuged (100,000 *g*, 20 min, 4 °C). The supernatant was discarded and the isolated pellet was gently resuspended in 1X SHE (100 μL 4 °C) and split into aliquots (i) frozen in N_2(l)_ for western blots and proteomics and(ii) resuspended in PBS (100 μL, 4 °C), mixed with 2x FGP fixative (100 μL, freshly prepared) and stored at 4 °C, protected from light, for electron microscopy. Continuous band B1 was similarly extracted, collected, and frozen in N_2(l)_ for Western Blot analysis.

To selectively eliminate organelle and vesicle contamination from MRO preparations, we conducted an additional procedure following all the standard steps described earlier (from whole cell to crude MROs, then MitoIP, followed by continuous density gradient), except for adding 0.3% digitonin to the cell homogenates immediately after nitrogen cavitation. The homogenate was then incubated on ice for 10 minutes at 4 °C, followed by low-speed centrifugation and subsequent high-speed ultracentrifugation to pellet crude MROs, consistent with the standard protocol outlined above.

#### Electron microscopy

##### Transmission electron microscopy (TEM)

Cell pellets or pellets of MRO of various purities were fixed in FGP fixative (see above, >2 h, rt), washed in 0.1 M cacodylate buffer, postfixed with 1% (w/v) osmium tetroxide (OsO_4_), 1.5% (w/v) potassium ferrocyanide (K_4_[Fe(CN)_6_]) (1 h, room temp). The samples were then washed twice in water and once in maleate buffer (50 mM maleate, pH 5.15) and incubated in 1% (w/v) uranyl acetate in maleate buffer (1 h, rt), followed by two washes in water and subsequent dehydration in increasing concentrations of ethanol (10 min each: 50%, 70%, 90%, twice in 100%). The samples were placed in propylene oxide for one hour and infiltrated overnight in a 1:1 mixture of propylene oxide and Spurr’s low viscosity resin (Electron Microscopy Sciences, Hatfield, PA). The following day, the samples were embedded in Spurr’s resin and polymerized (60 °C, 48 h). Ultra-thin sections (80 nm) were cut on a Reichert Ultracut-S microtome, collected on copper grids and stained with lead citrate, and examined in a JEOL 1200EX Transmission electron microscope, with images recorded using an AMT 2k CCD camera.

##### Quantification of MRO from transmission electron microscopy data

MRO sizes were quantified using transmission electron microscopy (TEM) images obtained from the sample of the highest purity (continuous gradient band B3) collected during the MRO isolation procedure. Representative low-power TEM fields were selected for quantitative analysis using NIH FIJI software. Using the Straight Line tool, each identified MRO’s length (longest dimension) and width (shortest dimension) were measured in nanometers. All measurements were calibrated according to the scale provided on the TEM images.

#### Antibodies and immunoblots

##### Generation of *Giardia* anti-GiOR-1 antibody

The full-length coding sequence of *Giardia lamblia* oxidoreductase 1 (GiOR-1, GL50803_0091252) was successfully cloned into a pET28b vector, featuring an N-terminal His-tag and SUMO fusion protein for enhanced expression and purification. This construct was transformed and overexpressed into *E. coli* NiCo21 (DE3) (New England Biolabs, Cat. #C2529H) cells (cultured in 2xYT broth at 37 °C with 225 rpm shaking) at an optical density of ~ 0.6. Protein expression was subsequently induced by adding 0.5 mM isopropyl-beta-D-1-thiogalactopyranoside (IPTG), followed by continued shaking for 16 hours at 18°C. Cells were harvested and lysed using a EmulsiFlexC50 in lysis buffer (50 mM Tris, pH 7.5, 300 mM NaCl, 10 mM imidazole, 10 mM β-ME) supplemented with 1 mM PMSF and 1 mM benzamidine. The lysate was then separated into soluble and membrane fractions via centrifugation (12,000 *g*, 30 min, 4 °C), with the soluble fraction proceeding to purification. Initial purification involved batch incubation with NiNTA resin for 1.5 hours at 4 °C, followed by transfer to a gravity column where the protein was washed with lysis buffer and eluted with an elution buffer (50 mM Tris pH 7.5, 300 mM NaCl, 300 mM imidazole, and 10 mM β-ME). To remove the His-tag, the eluted protein was incubated overnight with ULP1 in dialysis buffer (20 mM Tris, 150 mM NaCl, 10 mM β-ME). The dialyzed sample was then passed over a nickel column to remove His-SUMO particles, collecting the tag-free recombinant GiOR-1 in the flowthrough and wash steps. Final purification was achieved using size exclusion chromatography (SEC) on a Superose 6 increase 10/300 column (Cytiva) in a buffer (20 mM Tris, 150 mM NaCl, and 10 mM β-ME, pH 7.5). The purified GiOR-1 protein was then utilized as an antigen for custom antibody production in rabbits by Cocalico Biologicals, yielding initial and final bleeds of crude serum. The rabbit IgG was subsequently purified from the immunized rabbit serum using NAb^™^ Protein A Plus Spin column (Thermo Scientific^™^, Cat. # 89960), resuspended (1:1 v/v) with 50% glycerol, aliquoted, and stored at −20 °C for further use. For quality control, *Giardia* trophozoite samples were separated on 4–20% SDS-PAGE gel and subsequently transferred and immunoblotted using standard methods to verify the specificity of the custom anti-GiOR-1 antibody.

##### Generation of *Giardia* anti-Tom40 antibody

Potential target peptides within the *Giardia lamblia* Tom40 (GL50803_0017161) were identified by analyzing the commercially available human anti-Tom40 antibodies. A candidate peptide sequence, located on the cytosol-exposed beta loops of the N-terminus, was selected from the primary sequence and predicted 3D structure of *Giardia* Tom40. This target peptide sequence was provided to Biosynth Company for custom antibody production in rabbits. The resulting affinity-purified antibody was aliquoted and stored at −20 °C and −80 °C for long-term preservation. For quality control, *Giardia* trophozoite samples were separated on 4–20% SDS-PAGE gel and subsequently transferred and immuno-blotted using standard methods to verify the specificity of the custom anti-Tom40 antibody.

For Immunofluorescence imaging, the full-length coding sequence of *Giardia lamblia* Tom40 (GL50803_0017161) was cloned into the pET28a vector with N- and C-terminal His-tags. The construct was transformed into *E. coli* BL21-CodonPlus (DE3)-RIPL cells (Agilent, Cat. #230280) and grown at 37 °C to an OD600 of ~0.6. Protein expression was induced with 0.5 mM IPTG and continued for 4 h at 37 °C. Recombinant His-tagged Tom40 was purified under denaturing conditions using Ni-NTA affinity chromatography and subsequently buffer-exchanged and concentrated. Polyclonal antibody production was carried out in rabbits (Cocalico Biologicals), generating initial and final serum bleeds. IgG was purified from immune serum using Protein A agarose beads (Thermo Fisher, Cat. #20333) according to the manufacturer’s instructions, aliquoted, and stored at −20 °C for downstream applications.

##### Protein quantitation and immunoblots

For immunoblot analysis, frozen samples were thawed on ice (30 min at 4 °C) and resuspended in 1X RIPA buffer (150 mM NaCl, 50 mM Tris-HCl, pH 7.4, 0.25% deoxycholic acid, 1% NP-40, 1 mM EDTA). A 1:10 dilution of each sample was prepared using 1x RIPA buffer. BSA standards were prepared in the same buffer (1 x RIPA), and protein quantitation was performed using BCA assay according to the manufacturer’s instructions (microplate assay, Thermo Cat. #23225). All samples were normalized to equal protein amounts in 1X RIPA buffer and mixed with LDS loading buffer containing DTT (final concentration, 1x LDS, 10 mM DTT). Proteins were denatured by heating (30 min, 95 °C) and separated by SDS-PAGE (60 min, rt, 135 V) on a Tris-Glycine gel (Novex^™^ WedgeWell^™^, 1.0 mm, 4–20% Protein Gel) alongside a molecular weight marker (Thermo Fisher Spectra Multicolor Broad Range Ladder, Cat #26634). Proteins were subsequently transferred to a PVDF membrane (BioRad Trans-Blot Turbo, Mini, 0.2 um, Cat # 1704157). Membranes were washed in TBST (3 × 5 min, rt, gentle rocking), and blocked with EveryBlot Blocking Buffer (Bio-Rad, Cat # 12010020, 10 min, rt, gentle rocking). Rabbit primary antibodies against GiOR-1 (MRO matrix, 1:1000 v/v), Tom40 (MRO outer membrane, 1:1000 v/v), α-Tubulin (cytoskeleton, Cell Signaling Cat # 2125S; 1:1000 v/v), and HA-Tag (Cell Signaling Cat # 3724S; 1:3000) were prepared in blocking buffer. The membranes were incubated with primary antibody solutions overnight (4 °C, gentle rocking). After incubation, the membranes were washed with TBST (3 × 5 min, rt, gentle rocking) and subsequently incubated with HRP-conjugated secondary antibody (Cell Signaling Cat # 7074S, 1:10,000 v/v, 1 h, rt, gentle rocking). The membranes were then washed with TBST (3 × 5 min, rt, gentle rocking) and incubated with Clarity Western ECL Substrate (Bio-Rad, Cat # 1705061) (5 min, rt, gentle rocking) prior to imaging.

#### MRO proteomics

##### Preparation of samples for proteome analysis on mass spectrometry

Whole cell pellets and pellets of MRO of different purities were processed for mass spectrometry. Samples were lysed in SDS lysis buffer (5% SDS, 50 mM TEAB, pH 8.5, 2 mM MgCl_2_, 2 μg/mL Aprotinin, 10 μg/mL Leupeptin, 1 mM PMSF) and incubated (15 min, rt). To shear DNA, 3 μL of benzonase (250 units/μL, Sigma, E1014-25KU) was added to each sample, followed by thorough mixing. Insoluble content was pelleted by centrifugation (20,000 *g*, 10 min). The clear supernatant was transferred for S-Trap digestion, and its concentration was estimated using BCA protein assay. Disulfide bonds were reduced in 5 mM DTT and incubated (1 h, 25 °C, 1000 rpm shaking), and cysteine residues were then alkylated in 10 mM IAA (45 min, 25 °C, 1,000 rpm shaking in the dark). Finally, the samples were acidified by adding 12% (v/v) phosphoric acid at a 1:10 (acid: lysate, v/v) ratio.

Aliquots of 80–100 μg proteins per sample were processed using S-Trap Micro Columns (Protifi, C02-micro-40). Proteins were precipitated with at least 6x sample volume of ice-cold S-Trap buffer (90% methanol, 100 mM TEAB) and centrifuged (3,000 *g*, 1 min). Proteins were then washed 3 times with 150 μL S-Trap buffer and centrifuged (3,000 *g*, 1 min) and digested overnight at 25 °C with 20 μL digestion buffer (50 mM TEAB with trypsin and LysC, each at 1:50 enzyme:substrate w/w ratio). Specifically, the digestion buffer was passed through the column and added back onto the column. After overnight digestion, peptides were sequentially eluted by centrifugation (3,000 *g*, 1 min) with 40 μL each of 50 mM TEAB and 0.1% FA, and 2 × 40 μL of 50% ACN/0.1% FA. Eluates were snap-frozen in N_2(l)_ and dried by vacuum centrifugation.

The dry digested peptides were reconstituted in 500 μL of 1% FA and desalted using a Sep-Pak tC18 96-well Plate (40 mg, Waters^™^, SKU #186002320). The C18 wells were conditioned sequentially with 500 μL each of 100% ACN and, 50% ACN/0.1% FA, and 4 × 500 μL of 0.1% TFA. Peptides were then loaded onto the conditioned C18 wells and washed with 3 × 500 μL of 0.1% TFA, followed by 1 × 500 μL 1% FA. Desalted peptides were eluted twice with 500 μL of 50% ACN/ 0.1% FA. The eluates were snap-frozen in N_2(l)_ and dried by vacuum-centrifugation. The dry desalted peptides were reconstituted in 30% ACN/0.1% FA and quantified using a BCA assay.

##### LC-MS/MS analysis

Peptide samples were reconstituted in 3% ACN/0.1% FA for LC-MS/MS analysis. MRO proteomics were run on an Orbitrap Astral mass spectrometer (Thermo Scientific) coupled to a Vanquish Neo UHPLC system (Thermo Scientific). 500 ng peptides from each sample were injected onto an Aurora Elite TS C18 UHPLC column (PepSep C18 column (Bruker Daltonics, 25 cm × 75 um, 1.5 um particle size)) with a flow rate of 300 nL/min. Samples were run on a linear 60 minute gradient from 99% solvent A (0.1% FA), 1 min 3% solvent B (99.9% ACN, 0.1% FA), 46 min 22% B, 51.5 min 34% B, 52.5 min 50% B. MS1 spectra were measured with resolution of 240,000 at 200 m/z and a mass range from 300 to 1800 m/z. The maximum injection time for MS1 was set at 5 msec and with an AGC target of 2E6. MS1 spectra were acquired with a timed loop control of 0.6 sec. MS2 scans covered a mass range of 380–980 m/z with an isolation window of 4 Th. The maximum injection time was set to 5 msec for MS2 scans, with an AGC target of 5E5. Precursors were fragmented with higher-energy collisional dissociation (HCD) with a normalized collision energy (NCE) set to 27%.

##### Mass Spectrometry (MS) data processing

MS spectra were searched using Spectronaut 19. Data were searched against the GiardiaDB protein annotation (GiardiaDB-68_GintestinalisAssemblageAWB2019_AnnotatedProteins.fasta) in direct DIA mode using quantification settings with a precursor Q-value cutoff of 0.01, a precursor PEP cutoff of 0.2, protein Q-value cutoff (experiment) of 0.01, a protein Q-value cutoff (run) of 0.05, and a protein PEP cutoff of 0.75 with MS2 intensities used for quantitation. The MS/MS search included the fixed modification Carbamidomethyl (C), and variable modifications, Oxidation (M), Acetyl (Protein N-term), Deamidation (N), and FormylMet (Protein N-term), with a maximum variable modification count of 5.

Column sum normalization was applied across samples (normalized_intensity_g,s_ = scalingFactor * intensity_g,s_ /columnSum_s_, for protein group *g* and sample *s*, where columnSum_s_ is the sum of intensities of sample *s*, and scalingFactor = mean (columnSum_s_ for all *s*). Next, protein groups were expanded such that normalized_intensity_*p,s*_ = normalized_intensity_*g,s*_ for all proteins *p* in protein group *g*. Normalized intensities were log10-transformed and then MinProbAll imputation was performed (missing values were replaced with values drawn from a Gaussian with mean set to the 1^st^ percentile of values across all samples, and sigma set to the median of standard deviations of each protein across all samples).

##### Protein correlation profiling

Z-scores were computed for each protein across all samples from log10-transformed normalized_intensity values. The protein correlation profile (PCP) score was calculated as the Pearson correlation^26^ between the Z-score profile for each protein against the mean of three MRO markers: Tom40 (GL50803_0017161), IscU (GL50803_0015196), and Hspd1/Hsp60 (GL50803_00103891). Proteins with PCP score ≥0.95 were labeled “MitoViaPCP”.

##### Protein sequence and feature annotations

Protein sequences from GiardiaDB-68 (GiardiaDB-68_GintestinalisAssemblageAWB2019_AnnotatedProteins.fasta)^70,71^ were annotated with protein domains (HMMER version 3.1 using the Pfam-A database version 35)^72,73^, transmembrane helices (TMHMM version 2.0)^74^, and homology (BlastP version 2.2.30+)^75^ to *Homo sapiens* (RefSeq release 63, using the longest transcript per Entrez gene), and *Saccharomyces cerevisiae* S288C (downloaded from *Saccharomyces* Genome Database on 2023-05-22).^74^ Gene ontology annotations were downloaded from GiardiaDB (GiardiaDB-61_GintestinalisAssemblageAWB_GO.gaf) and mapped to the current genome assembly by identifier and then mapped to GO slim (current.geneontology.org/ontology/go.obo version 1.2, releases/2022-03-22). Figure 2B used GO slim cellular compartments (mapping nucleolus, chromosome, nuclear envelope to nucleus), excluding proteins assigned to multiple compartments.

##### Benchmarking accuracy

The “gold+” set of 32 known MRO proteins was created by literature curation of proteins with evidence of MRO localization based on: (i) epitope-tagging and microscopy showing co-localization with a MRO marker, or (ii) fractionation and Western blot with proper controls. Note that to avoid circularity, proteins linked to MRO only through mass spectrometry studies were not included in “gold+”. All publications linked to GO slim “mitochondria” cellular components in GiardiaDB-61 were manually reviewed, in addition to review articles and primary literature searches.

The “gold−” set of 928 non-MRO proteins was compiled via a union of two methods: 326 proteins with experimental evidence of non-MRO localization via GO slim (Method 1) union 642 proteins with orthologs to human and yeast non-mitochondrial proteins (Method 2). Method 1: all proteins with strong evidence of non-MRO cellular compartment (based on GO functional annotations with evidence codes EXP, IDA, TAS, IC that mapped to non-mitochondrial GO slim cellular compartments), excluding genes with any evidence of mitochondrial localization (annotations mapped to GOslim “mitochondria” with any evidence code), and excluding “gold+” literature annotations. Method 2: all *Giardia* proteins with orthologs (BlastP best bi-directional hit, E<0.001) to human and yeast non-mitochondrial proteins (n = 18,111 human RefSeq genes not in MitoCarta3.0^8^ and n = 4,753 yeast genes not in Morgenstern Cell Reports 2017 and lacking mitochondrial GOslim assignment in *Saccharomyces* Genome Database as of 2023-08-21).

We estimate that 1% of *Giardia* proteins (~50 proteins) are localized to the MRO. Based on this prior, we estimate “gold+” represents 64% of MRO proteins while “gold− “ represents only 19% of true non-MRO proteins, thus it is imperative to correct for the sizes of our benchmark sets when estimating false discovery rates. We benchmarked dataset accuracy using “gold+” and “gold− “ as described in (MitoCarta2.0)^76^: true positives (TP), true negatives (TN), false positives (FP), false negatives (FN), sensitivity SN = TP / (TP + FN), specificity SP = TN / (TN + FP), corrected false discovery rate cFDR = (1–SP)/(1–SP + SN x *O*_*prior*_), and Precision = 1 – cFDR, with *O*_*prior*_ = 0.01 prior probability of MRO localization.

#### Confocal immunofluorescence microscopy

##### Selection of microscopy targets

To test the localization of proteins in the MitoViaPCP list, 25 proteins across the full range of MitoViaPCP scores (1.0–0.95) were selected for HA-tagging and confocal immunofluorescence microscopy. These MitoViaPCP list targets included: 12 uncharacterized proteins with no predicted localization, and 13 gold+ MRO protein predictions. Outside of the MitoViaPCP list, 36 additional possible MRO candidates for tagging and microscopy were selected, prioritizing candidates using the following criteria: (i) MitoViaPCP score:0.95–0.84, (ii) known and validated MRO proteins (gold+) that scored low in our PCP analysis (n = 6) (iii) predicted gold negative proteins found in the MitoViaPCP list.

##### Cultures for microscopy

Wild-type or transfected *Giardia* trophozoite cells were cultured in 12.5 cm plug-seal cap tissue culture flasks (Fisher Scientific, Cat. # FB012934) in standard medium. The flasks were filled with media up to the neck to maintain anaerobic conditions and incubated (37 °C, stationary dry incubator) for 48–72 hours.

##### Fixation and immunostaining

For immunostaining, *Giardia* trophozoites were detached by incubating the flask on ice (30 min, 4 °C). The cells were collected, washed with 1x PBS, and immediately fixed with 4% paraformaldehyde (22 °C, 30 min). After fixation, the fixative was aspirated, and the cells were washed twice with PBS (22 °C, 1 min). The cells were then placed on PTFE printed slides (EMS, Cat. # ER-232W-72) and allowed to adhere. Once adhered, cells were permeabilized with 0.1% (w/v) Triton-X-100 in PBS (22 °C, 10 min) and blocked with 1% BSA in PBS (1 h, 22 °C). After blocking, primary antibodies were added (mouse anti-HA antibody (Cell Signaling, Cat. # 2367S, 1: 300) or mouse anti-Myc (Thermo Fisher Scientific, Cat. # 13-2500, 1: 500) and rabbit anti-Tom40 antibodies (custom-generated, 1: 500)) and then incubated for 1 h at room temperature. Following incubation, cells were washed three times with 1% BSA in PBS (~5 minutes each, 22 °C). Next, cells were incubated with fluorescently labelled secondary antibodies (Alexa Fluor 488, Thermo Fisher Scientific, Cat. #A11001, and Alexa Fluor 647, Thermo Fisher Scientific, Cat. # A21244, 1:500) for 1 h at room temperature in the dark. Following incubation, the cells were washed three times with 1% BSA in PBS (~5 minutes each, 22 °C), and the slides were mounted using VECTASHIELD Antifade Mounting Medium with DAPI (Vector Laboratories, Cat. # H-122-10) and a glass coverslip. The edges were sealed, and the slides were stored in the dark at room temperature.

##### Image acquisition and processing

Microscopy was performed using a Nikon Eclipse Ti2 and Nikon AX DUX-ST microscope equipped with laser lines at 405, 488, 561, and 640 nm. Z-series images were acquired, followed by 3D deconvolution using Nikon software. Background correction was applied using either NIS Elements AR version 6.10.01 or FIJI (ImageJ), and TIFF files of single planes from the Z-series were exported for further analysis.

#### MitoCarta analyses

##### MitoCarta Definition

The GlMitoCarta (n = 59) is the union of three lists: (i) MitoViaPCP (n = 38, PCP score ≥0.95), (ii) MitoViaMicroscopy (n = 46, localized to MRO by our confocal immunofluorescence microscopy), and (iii) Known MRO (n = 32, “gold+” literature). We assigned gene symbols to GlMitoCarta proteins with no known function including five previously known MRO proteins (MROX1, GL50803_008148; MROX2, GL50803_0017276; MROX3, GL50803_003491; MROX4, GL50803_007188; MROX5, GL50803_0012229;) and 27 new MRO proteins (MROX6, GL50803_0010182; MROX7, GL50803_0061303; MROX8, GL50803_005532; MROX9, GL50803_003202; MROX10, GL50803_0087492; MROX11, GL50803_0015569; MROX12, GL50803_0011701; MROX13, GL50803_0017298; MROX14, GL50803_0029682; MROX15, GL50803_002848; MROX17, GL50803_0095290; MROX16, GL50803_0016581; MROX18, GL50803_008651; MROX19, GL50803_0029692; MROX20, GL50803_0040863; MROX21, GL50803_0016677, MROX22, GL50803_0010148; MROX23, GL50803_006519; MROX24, GL50803_008657; MROX25, GL50803_0014161; MROX26, GL50803_008102; MROX27, GL50803_0015367; MROX28, GL50803_007135; MROX29, GL50803_0031617; MROX30, GL50803_003307; MROX31, GL50803_0014553; MROX32, GL50803_005859).

##### *In silico* structural analyses

Full-length protein sequences were analyzed for protein domains architecture using TMHMM 2.0 and DeepLoc 2.1 ^31,74^. Three-dimensional (3D) structures were retrieved from the AlphaFold database, and structural homologs were then identified using Foldseek^35^ by searching against both AlphaFold ^34,45^and PDB repositories^77^. Predictions of iron and iron-sulfur binding sites carried out using MIB2 (Metal Ion-Binding Site Prediction and Modeling Server)^38^ along with structural and sequence analyses. Remote homolog detection was performed through pLM-BLAST^36^ by sequence search against the ECOD70_20231201 structural database and HHpred analysis^37^ against the COG_KOG_v1.0 database, which includes bacterial and archaeal Clusters of Orthologous Groups (COG) as well as eukaryotic (KOG) entries.

##### Homology across species

Homology of *Giardia* proteins to 203 eukaryotic species, 29 bacterial clades (grouped at superfamily-level), and 5 archaeal clades (grouped at kingdom-level) was determined based on orthogroups that were inferred by OrthoFinder v2.5.5^78^, and refined using a custom pipeline incorporating profile Hidden Markov Model and Alphafold structure alignments (Chen *et al*.,^41^; also see michaelzhuchen GitHub).

##### Species tree

The species tree used for visualizations and phylogenetic presence/absence profiles was inferred from a concatenated alignment of universal ribosomal proteins across eukaryotes, bacteria, and archaea using a manually-refined NCBI taxonomy as a guide tree (Chen *et al*.,^41^; also see michaelzhuchen GitHub).

##### Determination of prokaryotic vs eukaryotic gene ancestry

The putative ancestry of each protein was assigned based on the presence/absence of its orthogroup across species. First, proteins with homologs in at least two prokaryotic clades were labeled as “Prokaryotic”. Second, proteins with homologs in at least two eukaryotic superfamilies (requiring homologs in at least two species within each superfamily) and in orthogroups containing >90% eukaryotic species were labeled as “Eukaryotic”. Third, proteins with no identified homologs beyond Metamonada were labeled as “Lineage-specific”. Lastly, any proteins that did not satisfy the criteria for these labels were labeled as “Indeterminate”.

#### Whole cell perturbation profiling

For whole cell perturbation profiling, *Giardia* trophozoites were first cultured under standard conditions as described above until they reached 60–70% confluency. Both adherent and non-adherent trophozoites were harvested and then resuspended in a small volume of standard medium for inoculation of the subsequent experimental cultures.

##### Metronidazole perturbation profiling cultures

To identify optimal concentration, preliminary experiments were carried out using metronidazole (Sigma, Cat. # M1547) at doses of 0, 2.5, 5, and 10 μM across various time points. Based on observed changes in cell morphology and viability, we selected a concentration of 10 μM and a treatment duration of 24 hours, as these conditions consistently caused significant morphological changes after drug exposure. A total of nine experimental cultures were used for metronidazole perturbation, with each of the three culture conditions repeated in biological triplicate in separate flasks: (i) control, (ii) vehicle control with DMSO, and (iii) metronidazole (10 μM). For metronidazole treatment, sealed plug-capped T-75 flasks containing 260 mL of media were inoculated with 4 × 10^7^ cells and incubated (24 h, 37 °C, stationary dry incubator). Light microscopy images of the culture flasks were captured immediately prior to harvest. Both adherent and non-adherent cells were collected. Adherent cells were detached by incubating the flasks on ice for 15 minutes at 4 °C. The media containing cells was transferred to 500 mL wide-mouth centrifuge bottles (Beckman Coulter, Cat. # 355607) and centrifuged (3,273 *g*, 10 min, 4 °C). The supernatant was carefully decanted and discarded. To recover any remaining cells, the flasks were rinsed with 50 mL of PBS containing 0.25 mM PMSF (a protease inhibitor); this wash was combined with the cell pellets in the centrifuge bottles, gently mixed, and centrifuged again (3,273 *g*, 10 min, 4 °C). The cell pellet was resuspended with PBS containing PMSF (1 mL, 4 °C), and a sample was further diluted for manual cell counting. The sample was aliquoted into 1.5-mL microcentrifuge tubes, centrifuged (5,500 *g*, 5 min, 4 °C), and the cell pellets were further processed as follows: (i) the cell pellet for protein analysis was immediately frozen in N_2(l)_ and then stored at −80 °C. (ii) the cell pellet for RNA analysis was resuspended in TRI reagent (500 μL, 4 °C) and then stored at −80 °C.

##### Iron perturbation profiling cultures

A total of six experimental cultures were established for iron perturbation profiling under two conditions: (i) standard media supplemented with ferric ammonium citrate (46 μM), and (ii) iron-depleted media (standard media without ferric ammonium citrate) supplemented with 300 μM deferoxamine, an iron chelator (Sigma, Cat. # D9533-1G). The experiment was performed in biological triplicate in separate flasks. Sealed plug-capped T-25 flasks (70 mL of media, final volume) were inoculated with 1 × 10^7^ cells and incubated (24 h, 37 °C, stationary dry incubator). Light microscopy images of the culture flasks were captured immediately before harvest. Both adherent and non-adherent cells were harvested. Adherent cells were detached by incubating the flasks on ice (15 min, 4 °C) and the content transferred to 50 mL Falcon tubes. The tubes were centrifuged (3,273 *g*, 10 min, 4 °C), supernatant was decanted and discarded. To recover any remaining cells, the flasks were rinsed with 15 mL of PBS containing 0.25 mM PMSF (a protease inhibitor); this wash was combined with the cell pellets in the Falcon tubes, gently mixed, and centrifuged again (3,273 *g*, 10 min, 4 °C). The pellet was resuspended with PBS (1 mL, 4 °C), and a sample was further diluted for manual cell counting by a hematocytometer. Samples for RNA analysis were resuspended in TRI reagent (500 μL, 4 °C) and then stored at −80 °C.

##### O_2_ perturbation profiling cultures

A total of 15 experimental cultures were established for O_2_ perturbation profiling across five different conditions: (i) anaerobic (<0.01% O_2_, 0 h), (ii) ambient air (21% O_2_) at three time points (1, 4, and 8 hours), and (iii) a de-oxygenation recovery condition (De-ox. (4h)), where cultures exposed to 21% O_2_ for 4 hours were returned to the anaerobic chamber to recover for an additional 4 hours. Each condition was performed in biological triplicate using separate flasks.

To culture *Giardia* trophozoites under strictly anoxic conditions, we used a glovebox anaerobic chamber (Coy Laboratory Products Inc.) and incubated the parasites for 24 hours at 37 °C in a dry, stationary incubator with a gas mix of 2.5% H_2_ and 97.5% N_2_. Cultures were initiated by inoculating T175 flasks (with vented caps to allow gas exchange) with 1 × 10^7^ cells in 30 mL of standard media. After 24 hours, cells were either collected immediately (0 h time point) or transferred to ambient air (21% O_2_) in a normal stationary dry incubator at 37 °C for different time points (1, 4, and 8 hours). To oxygenate the culture flask, the flasks were opened inside a biosafety hood and gently swirled to mix with the air. Following the 4 hours of 21% O_2_ exposure, one set of flasks was returned to the anaerobic chamber for deoxygenation recovery (De-ox. (4h)). Light microscopy images of the culture flasks were taken immediately before harvest. Both adherent and non-adherent cells were collected. To detach the adherent cells, flasks were incubated on ice for 15 minutes at 4 °C, and the content was transferred to 50 mL Falcon tubes. The tubes were centrifuged (3,273 *g*, 10 min, 4 °C), supernatant was decanted and discarded. To recover any remaining cells, the flasks were rinsed with 50 mL of PBS containing 0.25 mM PMSF (a protease inhibitor); this wash was combined with the cell pellets in the Falcon tubes, gently mixed, and centrifuged again (3,273 *g*, 10 min, 4 °C). The cell pellet was resuspended with PBS (1 mL, 4 °C), and a sample was further diluted for manual cell counting. For anaerobic conditions (<0.01% O_2_, 0 h), cells were harvested inside the anaerobic chamber using ice-cold PBS (50 mL, 4 °C). For brief centrifugation outside of the anoxic chamber, tubes were tightly capped and further sealed with parafilm. The sample was aliquoted into 1.5-mL microcentrifuge tubes, centrifuged (5,500 *g*, 5 min, 4 °C), and the cell pellets for RNA analysis were resuspended in TRI reagent (500 μL, 4 °C) and then stored at −80 °C.

##### Preparation of whole cell metronidazole perturbation profiling samples for proteomic analysis

Whole cell pellets were lysed by incubation on ice for 15 min in SDS lysis buffer (5% SDS, 50 mM TEAB, pH 8.5, 2 mM MgCl_2_, 2 μg/mL Aprotinin, 10 μg/mL Leupeptin, 1 mM PMSF). To degrade DNA, lysates were supplemented with 3 μL of 250 units/μL benzonase (Sigma, E1014-25KU), followed by thorough mixing. Lysates were then clarified by centrifugation (10 min, 20,000 *g*, 10 °C). The supernatant was collected, and protein concentration was determined by a BCA assay.

Disulfide bonds were reduced in 5 mM DTT (1 h, 25 °C, 1,000 rpm shaking). Subsequently, cysteine residues were alkylated in 10 mM IAA (45 min, 25 °C, 1,000 rpm shaking in the dark). The reaction was eventually quenched by adding 12% phosphoric acid at a 1:10 (v/v) ratio to the total volume (pH <3).

An equivalence of 80 μg proteins was aliquoted from each sample for digestion on S-Trap Micro columns (Protifi). Proteins were precipitated in 6x sample volume of ice-cold S-Trap buffer (90% methanol, 100 mM TEAB) by thoroughly mixing on the column. The mixture was centrifuged (3,000 *g,* 1 min). Proteins bound to the column were washed three times with S-Trap buffer, and on-column digestion was initiated by adding digestion buffer (50 mM TEAB with trypsin and LysC at 1:50 enzyme:substrate ratio, w/w). Specifically, the buffer was passed through the column with centrifugation (2,500 *g*, 1 min) and added back onto the column before incubating overnight at 37 °C. Then, peptides were eluted sequentially with 50 mM TEAB, 0.1% FA, and two elutions with 50% ACN/0.1% FA. Eluates of each sample were collected into the same tube, snap-frozen in liquid nitrogen, and vacuum-centrifuged to dry.

Dried peptides were reconstituted in 1% FA (pH < 3) and desalted using Sep-Pak tC18 96-well plates (40 mg; Waters, SKU #186002320). Wells were conditioned sequentially with neat ACN, 50% ACN/0.1% FA, and four washes with 0.1% TFA. Peptides were loaded, washed three times with 0.1% TFA and once with 1% FA, and eventually eluted twice with 50% ACN/ 0.1% FA. The eluates were snap-frozen in liquid nitrogen and vacuum-centrifuged to dry. Dried, desalted peptides were reconstituted in 30% ACN/0.1% FA, and peptide concentration was determined using a BCA assay.

##### LC-MS/MS analysis of perturbational profiles

Acidified peptides were loaded onto Evotips and prepared according to standard protocol. An EvoSep One coupled to a Bruker timsTOF HT mass spectrometer was used to elute the sample from the Evotip onto an Aurora Elite Column (IonOpticks). DIA-PASEF analysis was performed with DIA windows ranging from 330 m/z to 1523 m/z with 36 variable isolation windows and a cycle time of 1.67 s. Collision energy was ramped as a function of increasing mobility starting from 30 eV at 0.8 1/K0 [V-s/cm-2] to 55 eV at 1.6 1/K0 [V-s/cm-2].

##### Mass Spectrometry (MS) data processing

Mass spectrometry data were further searched and processed as described above in the MRO proteomics section.

##### RNA isolation for metronidazole, iron, and O2 perturbation profiling transcriptomics

For RNA isolation, the cell samples stored in TRI reagent were thawed on ice and resuspended by vortexing. After vortexing, the tubes containing the samples were centrifuged (15,000 *g*, 5 min, 4 °C) to pellet insoluble debris. The cleared lysate was then transferred to a fresh tube. RNA extraction was performed following the Direct-zol RNA Miniprep Plus protocol (Zymo Research, Cat. # R2071). An equal volume of ethanol (1:1, v/v; 500 μL) was added to the TRI reagent sample at room temperature and mixed by vortexing. The mixture was transferred to a Zymo-Spin IIICG column placed in a collection tube and centrifuged (15,000 *g*, 30 sec, rt). The column was then moved to a new collection tube, and the flow-through was discarded. DNase I treatment was performed on the column. Next, 400 μL of Direct-zol RNA PreWash was added to the column, followed by centrifugation (15,000 *g*, 30 sec, rt). The flow-through was discarded. This wash step was repeated once more with another 400 μL of PreWash buffer. Subsequently, 700 μL of RNA Wash Buffer was added to the column, centrifuged (15,000 *g*, 1 min, rt), and the flow-through discarded. To ensure complete removal of the wash buffer, the column was centrifuged again under the same conditions, and the flow-through was discarded. The column was then carefully transferred into an RNase-free tube. To elute the RNA, 50 μL of DNase/RNase-free water was added directly onto the column matrix and centrifuged (15,000 *g*, 30 sec, rt). RNA concentrations in samples were measured using a NanoDrop spectrophotometer, and the purified RNA was stored at −80 °C.

##### RNA-seq transcriptomics for whole cell perturbation profiling

RNA-seq transcriptomics performed for whole-cell perturbation profiling. RNA isolated from all three sets of perturbation experiments (metronidazole, iron, and O_2_) was submitted together for batch RNA-seq analysis (150 base pair paired-end reads) at Azenta.

##### RNA-seq data processing

The *Giardia intestinalis* genome assembly was obtained from GiardiaDB (GiardiaDB-68_GintestinalisAssemblageAWB2019_Genome.fasta). RNA-seq data were analyzed against the corresponding transcriptome, using annotated transcripts from “GiardiaDB-68_GintestinalisAssemblageAWB2019_AnnotatedTranscripts.fasta”. The raw sequencing reads were processed using a custom bulk RNA-seq processing pipeline. Paired reads were aligned to the *Giardia* genome (STAR options -- outSAMattributes NH HI NM MD AS XS nM, and – twopassMode Basic), duplicate reads were removed (Picard v 2.27.5, as distributed in GATK v 4.3.0.0, MarkDuplicates tool), reads were assigned to genes (featureCounts v 2.0.6, options -t exon -p, -M, --countReadPairs, and –primary), BAM format was created (SAMTools v 1.6), and read counts per gene were aggregated (featureCounts) to create a TPM matrix, then log-scaled (scanpy.pp.log1p function). Z-scores are displayed for visualization. Differential gene expression and adjusted P-values in Dataset S6 were calculated on the raw count data between each control (e.g., MTZ_con) and perturbation (e.g., MTZ-10uM) using the DESeq2 package (version 1.30.1).

#### Immunoprecipitation mass spectrometry (IP-MS) analysis

For IP-MS analysis, *Giardia lamblia* wild-type trophozoites, or trophozoites overexpressing 3xHA-tagged MRO proteins (i.e., MOMP35, MOMTiP-1, MOMTiP-4, MROX6, MROX13 and MROX14) were cultured under standard conditions until they reached 60–70% confluency. Both adherent and non-adherent trophozoites were harvested and resuspended in a small volume of standard medium for inoculation of the subsequent experimental cultures.

##### Crude MRO preparation

Crude MROs from *Giardia lamblia* strain WB, wild type (WT), and transgenic lines expressing 3xHA tagged MRO proteins were maintained in log phase growth in standard media for three days prior to the experiment. The experimental cultures (175-cm plug-seal flasks, 700 mL standard medium) were inoculated with 1×10^7^ trophozoites and incubated (37 °C, stationary, 48–72 h) until cells reached confluency and crude MROs were prepared as described above. In brief, both adherent and non-adherent cells were harvested, pelleted by centrifugation (3,000 *g*, 10 min, 4 °C), washed and resuspended in 1X SHEP. Following cell harvesting, cells were mechanically lysed by nitrogen cavitation (1100 PSI, 15 min, 4 °C, 2 cycles), and the cell homogenate was subjected to differential centrifugation (10,000 *g*, 15 min, 4 °C, 2 cycles) to pellet intact cells and nuclei. The slow-spin supernatant was transferred to a 38.5-mL Beckman Coulter ultracentrifuge tube (25 × 89 mm, Ultra-Clear, Cat. # C14292) and then ultracentrifuged (SW 32 Ti rotor, 100,000 *g*, 30 min, 4 °C) to recover a crude MRO pellet. The high-speed supernatant was aspirated and disposed. The resulting pellet was mixed with the 1xSHE buffer, aliquoted and immediately frozen in N2(l).

##### Co-immunoprecipitation (Co-IP)

Co-Immunoprecipitation (Co-IP) experiments were performed using anti-HA magnetic beads on crude MRO fractions derived from WT cells and transgenic lines expressing 3xHA tagged MRO proteins. Lysis and IP conditions were pre-optimized for each line, including buffer composition, protein-to-bead ratio, and sample-bead incubation time. Based on the optimized sample-bead incubation times, experiments were conducted in three batches, each in biological duplicate and paired with a corresponding WT control to ensure comparable conditions. Batch 1 included WT1, MOMP35, and MROX6 (2 h incubation at 4 °C); Batch 2 included WT2, MOMTiP-1, and MOMTiP-4 (4 h at 4 °C); and Batch 3 included WT3, MROX13, and MROX14 (overnight incubation at 4 °C).

Crude MRO pellets were thawed on ice (30 min, 4 °C) and solubilized in 1x lysis buffer (50 mM Tris-HCl, pH 7.4, 150 mM NaCl, 0.25% deoxycholic acid, 1% NP-40, 1 mM EDTA, Roche Complete protease inhibitor, 500 μM PMSF) at a 1:20 dilution. Samples were incubated (laid on ice for 10 min), then briefly vortexed and centrifuged (15,000 *g*, 10 min, 4 °C) to collect the supernatant. Protein concentration was determined by BCA assay per standard protocol (microplate assay, Thermo Cat. #23225). IP reactions were performed in 1.5 mL Eppendorf tubes in a final volume of 0.9 mL containing ~350 μg of crude MRO protein and 50 μL anti-HA magnetic beads (Pierce, Cat. #88837). Beads were pre-washed twice with 1x lysis buffer prior to use. Reactions were incubated at 4°C (cold room) with gentle rotation and incubation time varied by bait protein used (Batch 1: 2h, Batch 2: 4h and Batch 3: overnight). Following incubation, the tubes were placed on a magnetic separation rack (Cell Signaling, #7017) and unbound material was removed. Beads were washed twice with 900 μL of Wash Buffer I (9:1 mixture of 150 mM NaCl, 50 mM Tris (pH 7.5), Roche Complete protease inhibitor; and 1x lysis buffer) by incubation for 4 min, at 4 °C with gentle rotation followed by magnetic separation and buffer removal using magnetic rack. The beads were subsequently washed once with 900 μL of Wash Buffer II (150 mM NaCl, 50 mM Tris, pH 7.5) and twice with 900 μL of Tris-Buffered Saline (TBS; 75 mM NaCl, 50 mM Tris-HCl, pH 8), by incubation each for 4 min, at 4 °C, with gentle rotation followed by magnetic separation and buffer removal using magnetic rack.

After the final wash, the beads were resuspended in 50 μL TBS and then immediately snap frozen in N_2(l)_ for downstream proteomics. For quality assessment, 5% of samples (45 μL) was collected at each step and analyzed by western blotting. Bead-bound proteins were eluted in 60 μL of 1x LDS by heating at 95 °C for 10 min, briefly vortexed, and separated from beads using a magnetic rack. Western blotting was performed to confirm efficient bead binding during immunoprecipitation and enrichment of HA-tagged proteins in the final eluate prior to proteomics analysis.

##### LC-MS/MS analysis of IP-enriched fractions

Magnetic beads bound with the respective *Giardia* 3xHA tagged MRO and Co-IP proteins were washed four times with 200 μL of 50 mM Tris-HCl (pH 7.5), then incubated in 100 μL of urea digestion buffer (2 M urea, 50 mM Tris-HCl pH 7.5, 1 mM DTT, 0.4 μg trypsin) at 25 °C for 1 h with shaking at 1000 rpm, and the eluate was subsequently transferred to a fresh tube. Beads were subjected to a second round of digestion in another 100 μL of urea digestion buffer at 25 °C for 30 min with shaking at 1000 rpm, and the eluate was pooled with the first. Beads were then washed twice with 60 μL of urea wash buffer (2 M urea, 50 mM Tris-HCl pH 7.5), and the washes were combined with the pooled eluate.

The combined eluate was reduced with 4 mM DTT for 30 min at 25 °C with shaking at 1000 rpm, then alkylated with 10 mM iodoacetamide (IAA) for 45 min at 25 °C with shaking at 1000 rpm in the dark. Samples were digested overnight with an additional 0.5 μg of trypsin at 37 °C with shaking at 800 rpm. Digests were acidified to 1% formic acid (pH < 3) using neat formic acid.

LC-MS/MS was performed as described above in the perturbational profiling section.

##### Mass Spectrometry (MS) data processing

Mass spectrometry data were further searched and processed as described above in the MRO proteomics section.

#### Yeast complementation analysis

##### Yeast strains

Complementation experiments were performed using five single knockout (*aac2*Δ, *leu5*Δ, *mir1*Δ, *mtm1*Δ, *sam5*Δ) and two double knockout (*ndt1*Δ*ndt2*Δ*, mrs3*Δ*mrs4*Δ*)* yeast (*S. cerevisiae*) SLC25A strains. Wild type (WT) and knockout yeast strains were derived from the W303a background (*MATa leu2-3 leu2-112 trp1-1 can1-100 ura3-1 ade2-1 his3-11*). Knockout strains were generated by PCR-based homologous recombination, where ORFs of the gene of interest were replaced by the *TRP1* deletion cassette. All strains used in this study are listed in the key resource table.

##### MOMTiP-4 expression and complementation

For complementation studies, codon-optimized *Giardia* MOMTiP-4 (MOMTiP-4-HA) was synthesized and cloned under the constitutive TEF1 promoter into a pRS405 vector containing *LEU2* as a selectable marker to generate the pTEF1-MOMTiP-4-HA-*LEU2* construct. Empty vector (pRS405-TEF-*LEU2*) or pTEF1-MOMTiP-4-HA-*LEU2* construct was linearized and transformed into yeast strains by the lithium acetate/PEG4000 method. Transformants were selected and maintained on complete supplement mixture (CSM: 0.5% Ammonium Sulfate, 0.17% yeast nitrogen base without amino acids and 2% glucose) lacking leucine.

Following transformation, western immunoblotting was performed to detect the expression and mitochondrial localization of *Giardia* MOMTiP-4-HA. WT yeast cells expressing MOMTiP-4-HA or an empty pRS405 vector were grown in CSM-Leu with 2% glucose overnight at 30°C, and back-diluted into 50mL fresh CSM-Leu media and grown to the stationary phase. Cells were harvested, and mitochondria were purified as previously described^79^. Spheroplasts were prepared by zymolyase (Zymo Research, Cat no #E1004) treatment (1 hour at 30°C with gentle shaking) and homogenized by Teflon homogenizer (15 strokes). Protein concentration was measured by BCA protein assay. Equal amounts of protein (50 μg) from whole cell lysates or mitochondrial fractions were analyzed by immunoblot analysis using anti-PGK1 (cytosol, Thermo Fisher Cat # PA5-28612, 1:1000 dilution in EveryBlot Blocking Buffer), anti-MTCO1 (mitochondria, Abcam Cat # ab110270, 1:1000 dilution in EveryBlot Blocking Buffer), and anti-HA-Tag antibodies (Thermo Fisher Cat#26183, 1:2000 dilution in EveryBlot Blocking Buffer). For spot assays, yeast cells were grown to the stationary phase in CSM-Leu liquid media containing 2% glucose. Samples were then diluted to a final OD_600_ of 0.1 before being serially diluted (1:5 successive dilutions) in water and spotted onto CSM-Leu plates containing either 2% glucose or 3% glycerol using a multi-pin replicator. Plates were incubated at 30°C for 3 days (glucose) or 5 days (glycerol) prior to imaging. Images were representative of 3 biological replicates (n = 3) per condition.

##### Mitochondrial iron measurement

For quantitative measurement of iron content, yeast cells (WT, *mtm1*Δ and *mtm1*Δ+MOMTiP-4-HA) were grown in YPD medium (1% yeast extract, 2% peptone, 2% glucose) to late exponential phase and harvested by centrifugation at 3,000 × g for 5 minutes. Mitochondria were isolated as described above^79^ and total iron content was measured using ferrozine colorimetric assay as previously described ^80^. Briefly, mitochondrial aliquots and FeCl_3_ standards were mixed with 10 mM HCl and freshly prepared iron-release reagent (1.4 M HCl and 4.5% w/v KMnO_4_) followed by incubation at 60°C for 2 hours in a fume hood. After cooling to room temperature, the samples/standards were centrifuged at 10,000 *g* for 5 minutes to remove insoluble debris. The supernatant was then incubated with iron detection reagent (6.5 mM ferrozine, 6.5 mM neocuproine, 2.5 M ammonium acetate, and 1 M ascorbic acid) for 30 minutes at room temperature. Iron content was determined by measuring absorbance at 550 nm and normalized to mitochondrial protein content. The experiment was performed three times (independent biological replicates). Data are normalized to wild-type (WT) control and presented as fold changes relative to WT (n=3). Statistical significance was computed using a paired Student’s t-test, performed using Graphpad Prism software.

### QUANTIFICATION AND STATISTICAL ANALYSIS

Statistical analyses were performed in Excel and R. Details of the benchmark analysis of published and newly generated MRO proteomics datasets are provided in the Methods and include how benchmark datasets were created, corrected for size, and used to determine the sensitivity and precision of the MitoViaPCP list and previously published lists. Two-sided Student’s t-tests (shown in volcano plots and barplots in the figures) were performed using the function t.test in Excel on input data that met assumptions of normal distribution. Enrichment analyses were performed using Gene Set Enrichment Analysis^81^ and Fisher’s Exact tests as described, using pre-ranked lists, reporting q-values (corrected for multiple-hypothesis testing), and replacing q = 0 with q<0.0001 based on the number of permutations specified.

Statistical analysis of IP-MS samples was performed using the Proteomics Toolset for Integrative Data Analysis (Protigy, v1.0.7; Broad Institute, https://github.com/broadinstitute/protigy). MS2 intensities were log2-transformed and median-normalized before a 2-sample moderated t-test was performed. P-values associated with every protein were adjusted using the Benjamini-Hochberg FDR approach.

## Supplementary Material

1 **Data S5. IP-MS analysis of tagged MRO proteins. Related to** Figure 5. Log2-transformed, median-normalized protein abundances measured across three IP-MS batches (9 experimental conditions with 2 biological replicates each; n = 18 samples).

2 **Data S1. Mass spectrometry (MS) proteomic of MRO samples. Related to** Figure 2. (A) DIA-MS intensity and iBAQ values for protein groups and (B) peptides corresponding to the MRO isolation from wild type (Workflow 1, continuous density gradient) and MOMP35-3xHA transfected (Workflow 2, MitoIP +continuous density gradient) *Giardia lamblia* parasites [14 samples (wild type) +14 samples (MitoIP)=28 samples].

3 **Data S2. MRO Protein Correlation Profiling (PCP) analysis. Related to** Figure 2. (A) Protein Correlation Profiling (PCP) results for all *Giardia* genes, (B) the MitoViaPCP list, and (C) benchmark statistics of MitoViaPCP.

4 **Data S3. *Giardia* MRO proteome inventory. Related to** Figure 3. (A) GlMitoCarta list of MRO proteins, and (B) non-MRO candidates.

5 **Data S4. Orthologous group (OG) analysis. Related to** Figure 4. (A) Orthologous group (OG) assignment for *Giardia*, human, yeast, and *Arabidopsis,* including all OGs, and (B) MitoCarta OGs.

6 **Data S6. Perturbation transcriptomics. Related to** Figure 5. RNA-seq expression data for metronidazole perturbation (3 conditions × 3 replicates = 9 samples), iron perturbation (2 conditions × 3 replicates = 6 samples), and O_2_ perturbation (5 conditions × 3 replicates = 15 samples) (total of 30 samples).

7 **Data S7. MS proteomics of metronidazole perturbation. Related to** Figure 5. (A) DIA-MS Intensity and iBAQ values for protein Groups and (B) peptides corresponding to the metronidazole perturbation experiment (3 conditions × 3 replicates = 9 samples), as well as (C) a gene-based summary.

9 **Table S1. MitoCarta Tree of Life Consortium.** This table lists the names and contact details of the authors who are part of the MitoCarta Tree of Life Consortium.

8 Document S1. Figures S1–S7.

## Figures and Tables

**Figure 1.**
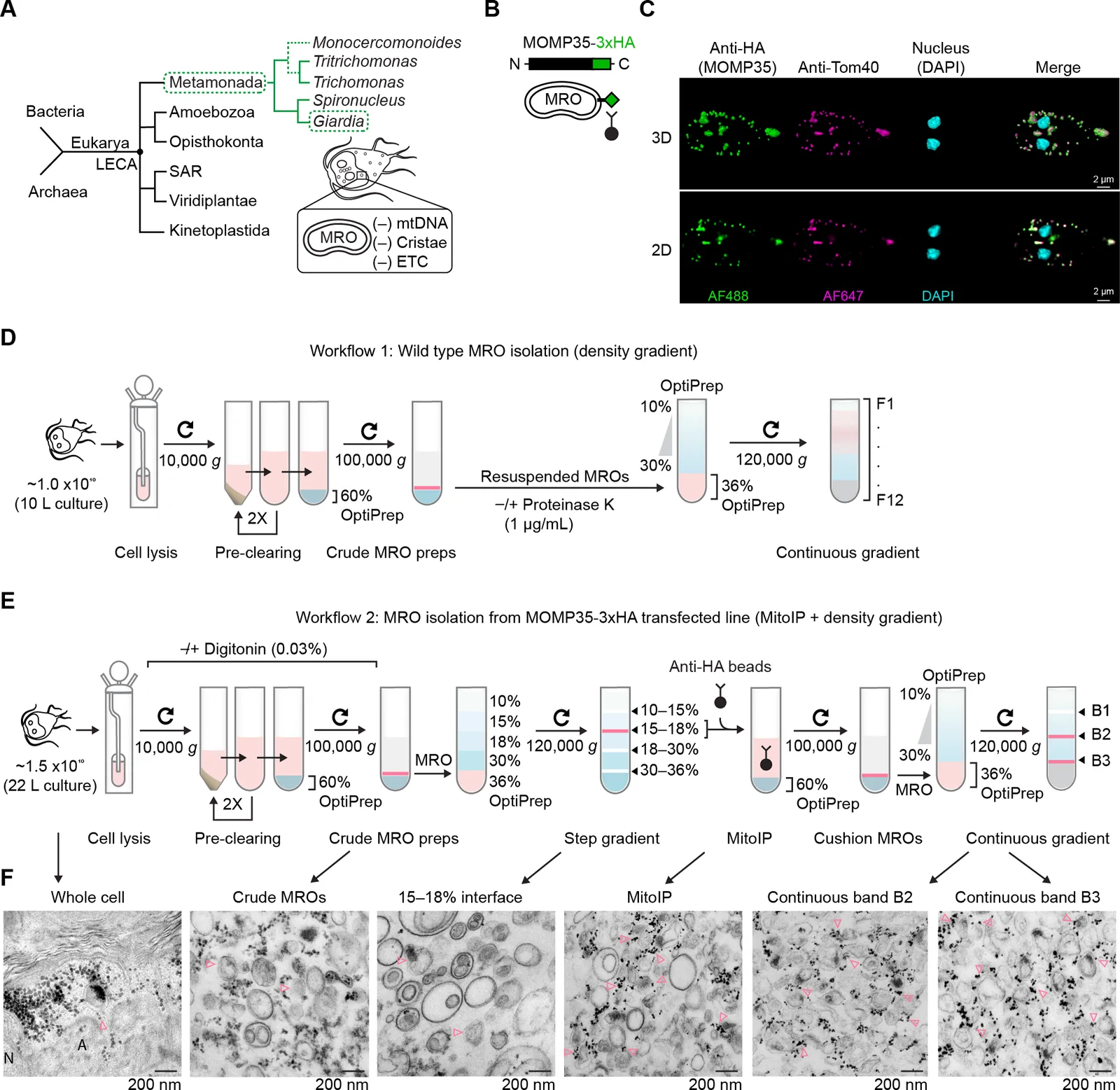
Serial density fractionation and affinity purification enriches *Giardia* MROs. (A) Phylogenetic placement of *Giardia lamblia* within the tree of life, highlighting its relationship to other domains and clades, particularly Metamonada, which have undergone reductive evolution resulting in the loss (*Monocercomonoides*) or extreme reduction of mitochondria. (B) Construct design (MOMP35-3xHA) used for isolation of MROs by mitochondrial immunoprecipitation (MitoIP) using anti-HA magnetic beads targeting the C-terminus 3xHA tags (see Methods). (C) Confocal immunofluorescence images of *Giardia* trophozoites expressing the MOMP35-3xHA construct. (D) Workflow 1 schematic of MRO isolation from wild-type *Giardia* trophozoites via differential centrifugation followed by continuous density gradient fractionation. (E) Workflow 2 schematic of MRO isolation from transfected parasites (MOMP35-3xHA) using differential centrifugation, step-gradient fractionation, MitoIP, followed by continuous density gradient fractionation. (F) Transmission electron microscopy (TEM) images depicting MRO samples at successive purification stages from Workflow 2 (E) as indicated by arrows: whole cell, crude MROs, 15–18% step gradient interface, MitoIP, and continuous density band B2 and B3. The pink arrowheads indicate either MROs (whole cell, crude MROs, 15–18% interface) or MROs bound to magnetic beads (Black dots in MitoIP, Continuous Band B2 and B3). See also Figure S1.

**Figure 2.**
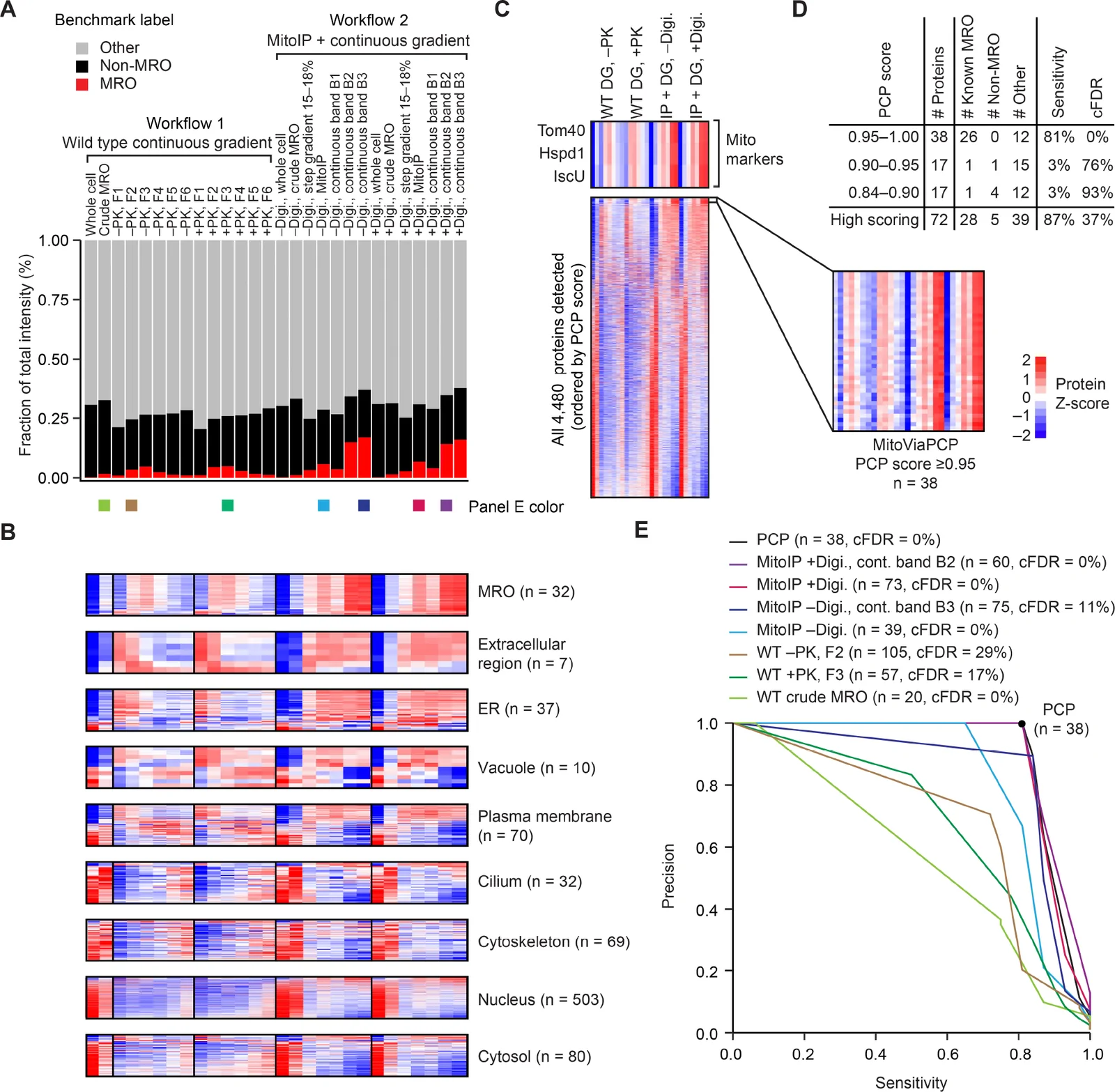
Mass spectrometry and protein correlation profiling (PCP) of *Giardia* MRO. (A) Stacked bar plot displays total mass spectrometry (MS) intensity of samples derived from both workflow 1 and workflow 2, colored by benchmark labels. (B) Heatmaps show relative MS abundance of proteins (rows) across all 28 samples from panel A (columns), with genes grouped by cellular compartment from MRO (literature, gold+) or GiardiaDB Gene Ontology. (C) Heatmaps show relative MS abundances of proteins (rows) across all 28 samples (columns), for all detected proteins (bottom panel), sorted by correlation with the average of three highly conserved mitochondrial marker proteins (top panel). Inset heatmap shows proteins with PCP score ≥0.95. (D) Accuracy of PCP scores of different thresholds, based on benchmark dataset. (E) Sensitivity and precision curves derived from benchmark data for PCP (black line) and log_2_(sample/whole cell) metrics for selected samples (colors corresponding to boxes beneath Panel A), including the number of proteins identified and the corresponding cFDR. See also Figure S2, Data S1 and S2.

**Figure 3.**
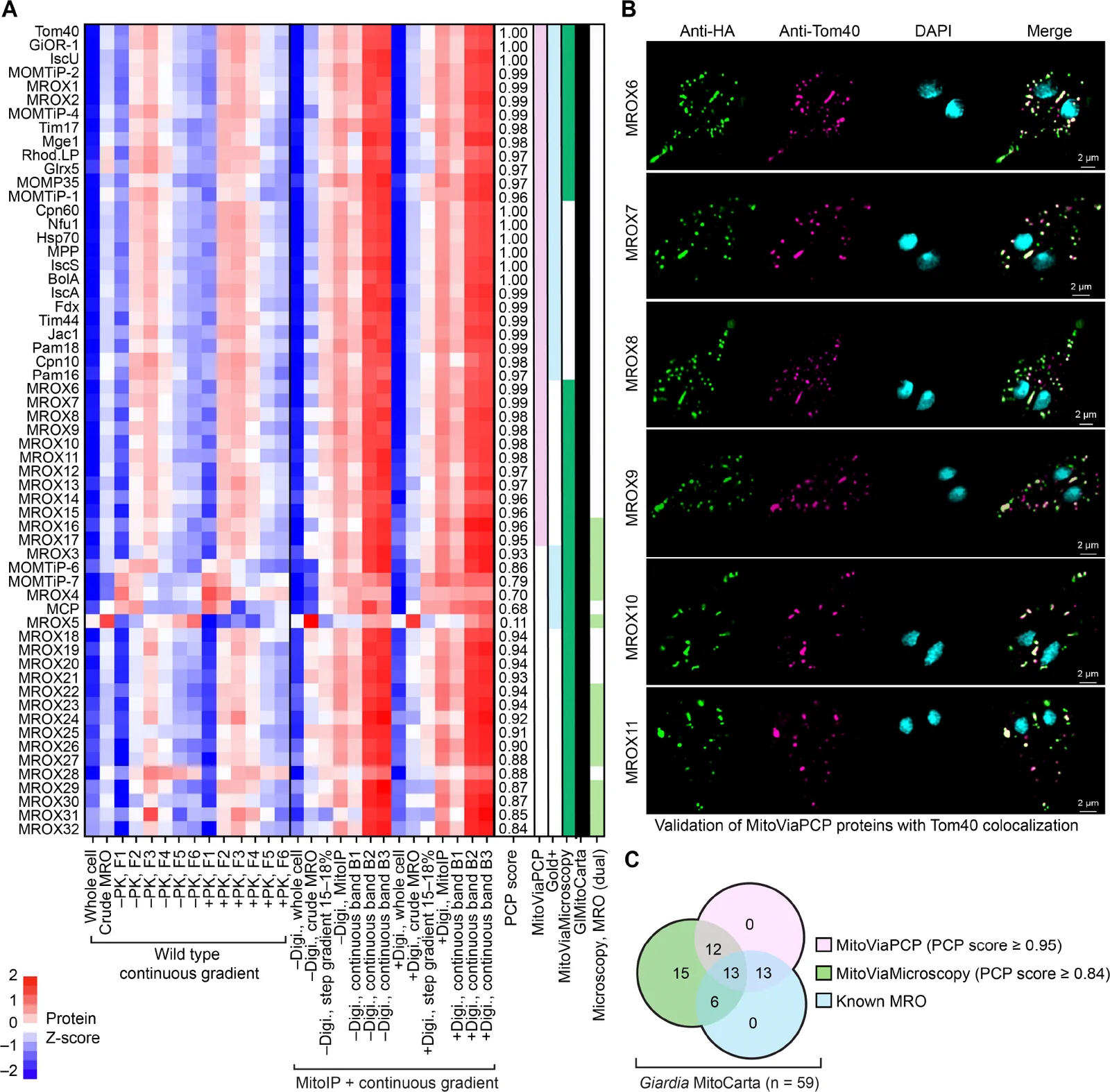
*Giardia* MRO proteome (GlMitoCarta). (A) Heatmap shows relative mass spectrometry (MS) abundance of GlMitoCarta proteins (n = 59). Colored bars at right classify proteins as MitoViaPCP (PCP scores ≥0.95, pink), gold+ (literature-based MRO evidence, light blue), MitoViaMicroscopy (MRO localization validated by HA-tagging and microscopy, dark green), GlMitoCarta (black), and Microscopy, MRO (dual) (microscopy validation of localization to MRO and other subcellular structures, light green). (B) Confocal immunofluorescence microscopy validation of top-scoring novel MRO proteins (PCP ≥0.97). Anti-HA antibody labeled HA-tagged proteins (Alexa Fluor 488, green), anti-Tom40 antibody mark endogenous Tom40 and MRO structures (Alexa Fluor 647, magenta), and DAPI labeled nuclear DNA (cyan). (C) Venn diagram shows composition of the *Giardia* MRO proteome (GlMitoCarta) created as the union of MitoViaPCP, literature (gold+), and microscopy validation (MitoViaMicroscopy). See also Figures S3 and S4, Data S3.

**Figure 4.**
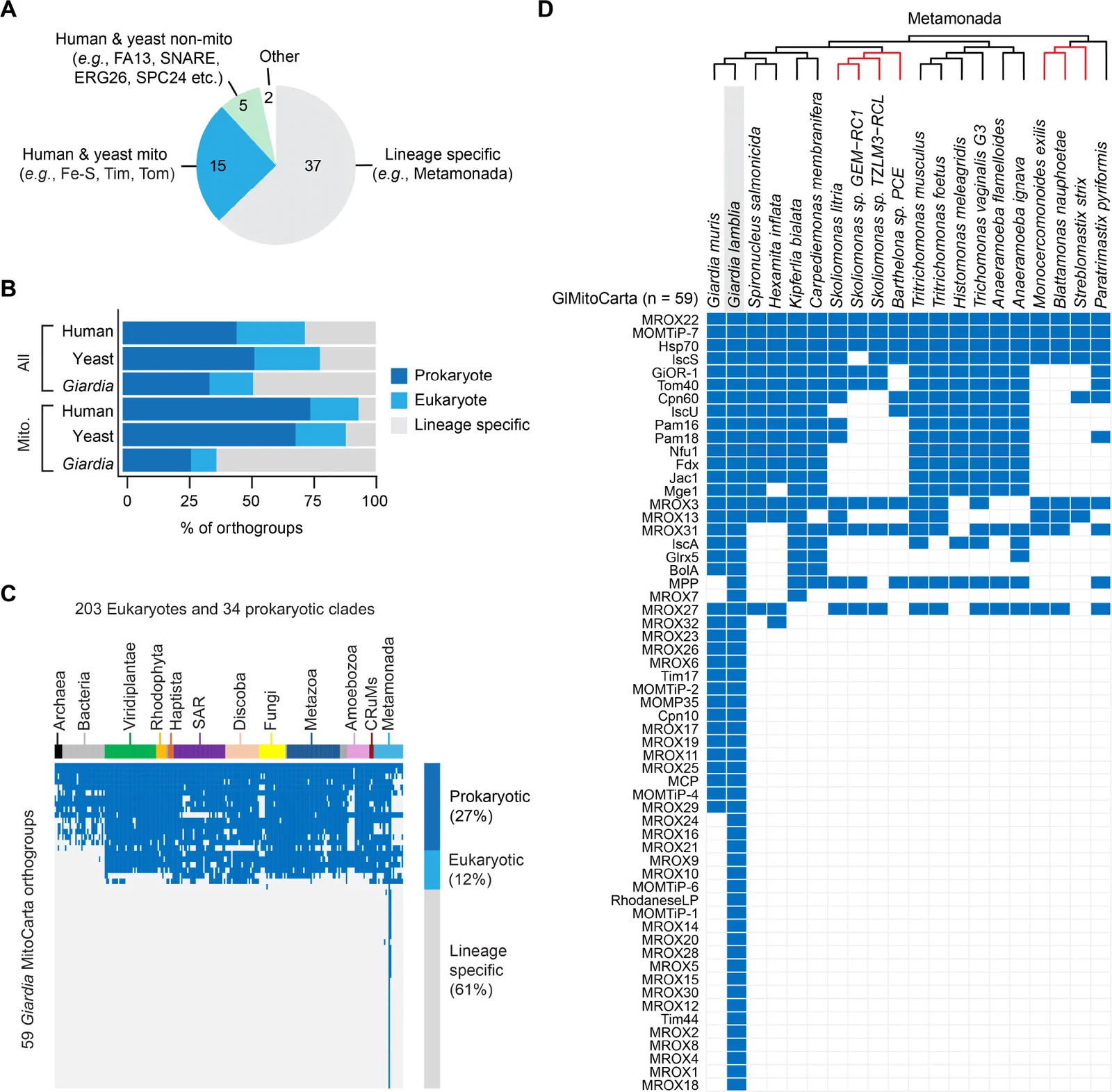
Evolutionary analysis of *Giardia* MRO proteins. (A) Pie chart of 59 GlMitoCarta proteins shows orthogroup memberships with mitochondrial proteins of humans^8^ or yeast^9^. (B) Comparison of MRO protein homology relationships to those of all proteins and mitochondrial proteins from human and yeast (based on orthogroup membership; lineage indicates Opisthokont or Metamonada clades). (C) Presence and absence matrix of GlMitoCarta proteins (rows) across a range of 203 eukaryotic species and 34 prokaryotic clades (columns). Blue squares indicate shared orthogroup membership. The homology label to the right indicates the presence of orthologs in prokaryotes, eukaryotes, or limited to Metamonada. (D) Presence and absence matrix of GlMitoCarta proteins (rows) across 20 Metamonada species. Blue squares indicate shared orthogroup membership. Red branches suggest a potential loss of MRO, indicated by significant absences of MRO proteins. The occasional absence of homology may be due to poor genome annotation or extreme sequence divergence. See also Figure S5 and Data S4.

**Figure 5.**
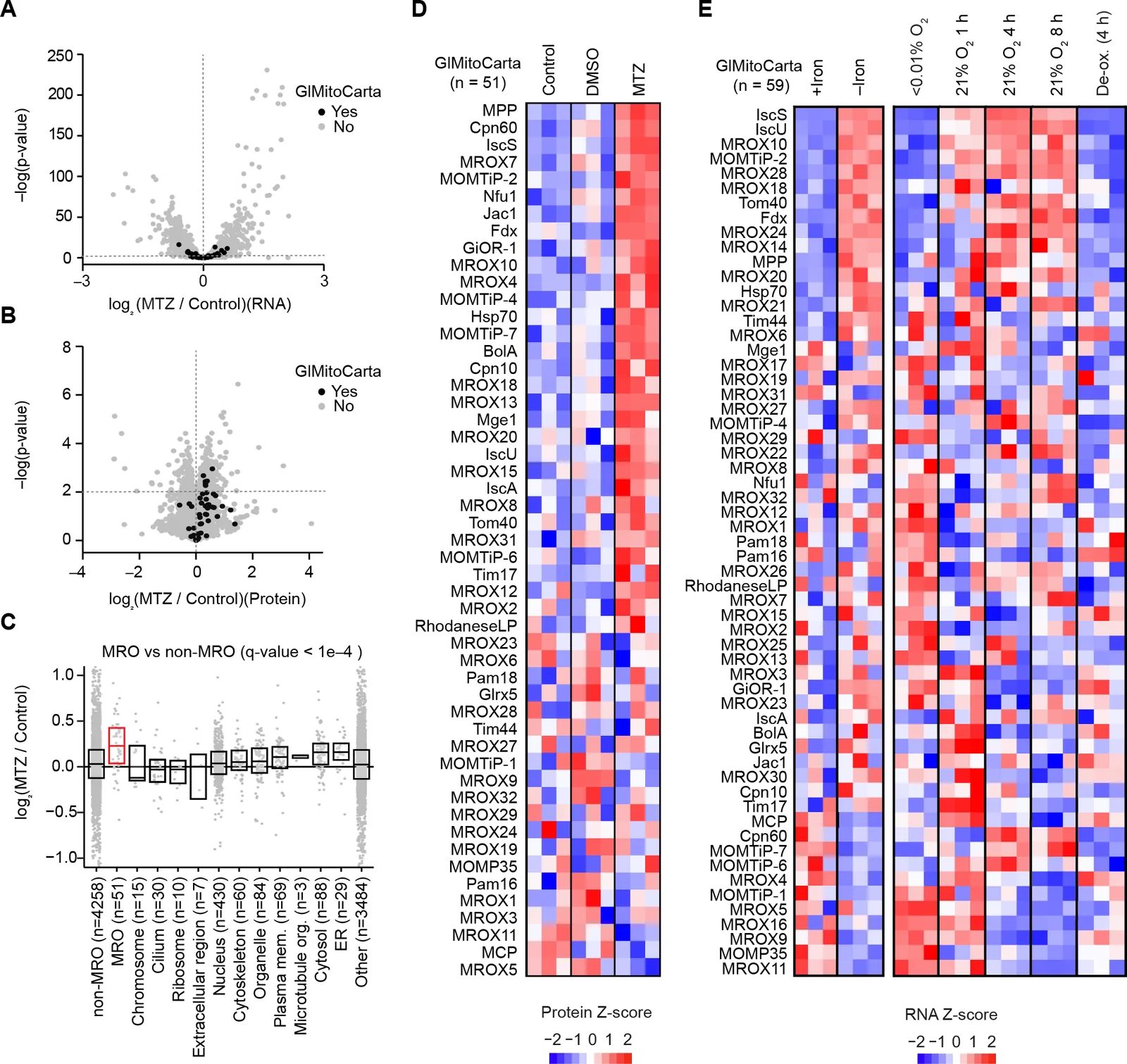
*Giardia* MRO proteome responds to metronidazole (MTZ) and environmental perturbations (iron depletion and O_2_ exposure), reinforcing the central role of the ISC pathway. (A) Volcano plot shows log_2_(MTZ /control) versus significance (DESeq2 p-value) for RNA-seq transcripts of all detected genes. Black dots represent the MRO genes, while grey dots are non-MRO genes. The dashed horizontal line indicates p-value <0.01. (B) Volcano plot of log_2_(MTZ/Control) versus significance for all detected proteins. Black dots represent MRO proteins, while grey dots are non-MRO proteins. The dashed horizontal line indicates p-value <0.01. (C) Boxplots show the abundance distribution of MRO and non-MRO proteins (25^th^-75^th^ percentile), along with individual proteins (dots) across 12 pathways. q-value represents FDR from GSEA comparing the MRO versus all proteins. (D) Heatmap displays relative abundances (protein Z-score) of 51 detected GlMitoCarta proteins across the metronidazole perturbation. (E) Heatmaps display relative transcript abundances (RNA Z-score) of 59 GlMitoCarta genes across the iron perturbation (−iron/+iron) and separately the O_2_ perturbation time points (0 h, 1 h, 4 h, 8 h, and 4 h De-ox. (4h)). See also Figure S6 and Data S5, S6, and S7.

**Figure 6.**
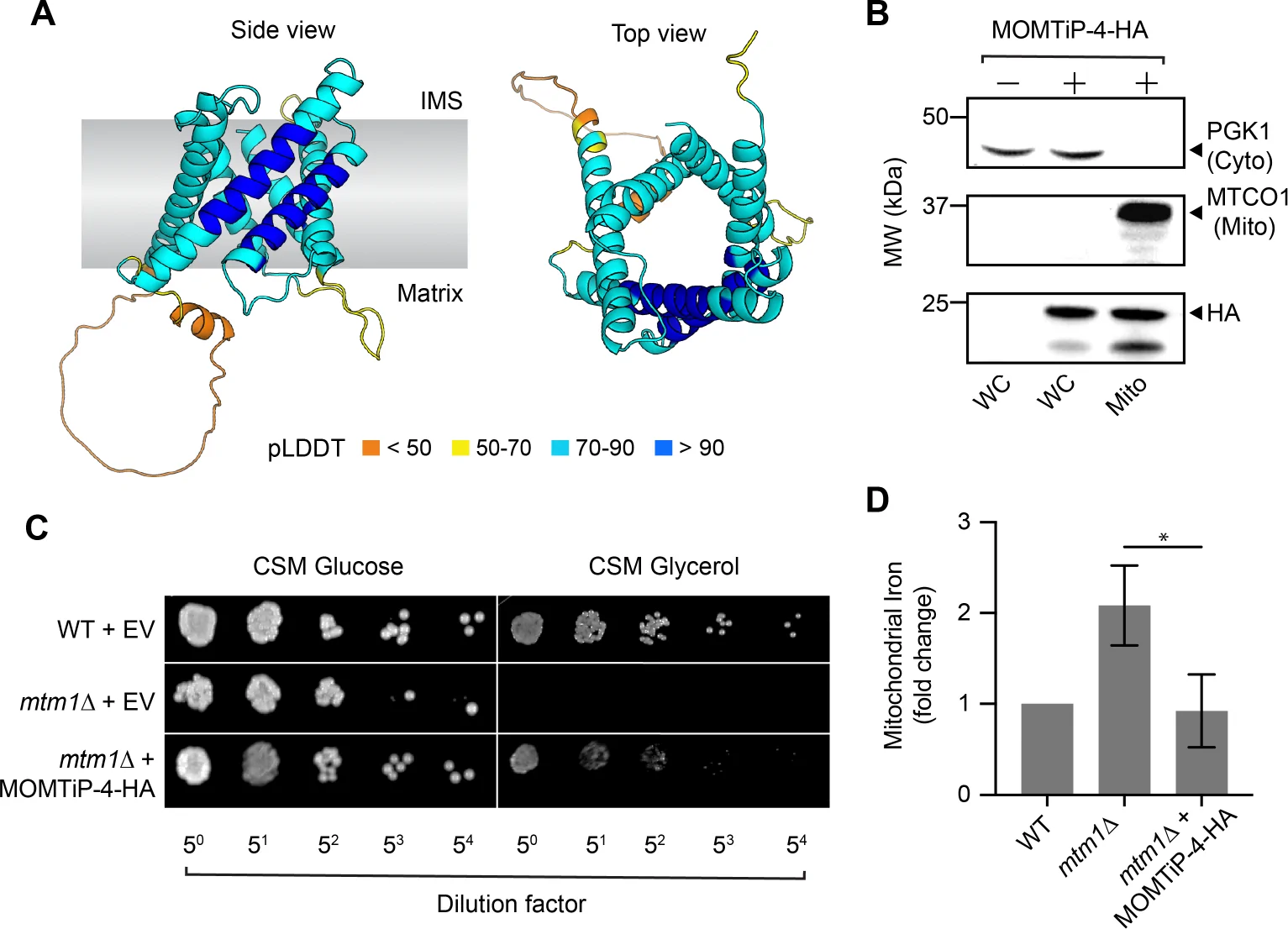
MOMTiP-4 functionally complements yeast *MTM1* (SLC25A) solute carrier. (A) AlphaFold3 predicted structure of MOMTiP-4, showing six α-helices with pseudo-symmetry, resembling the SLC25A family of transporters (colored by predicted local distance difference test (pLDDT)). (B) Immunoblot of whole-cell lysates (WC) and purified mitochondria (Mito) from wild-type yeast cells expressing either an empty vector or codon-optimized HA-tagged MOMTiP-4. (C) Serial dilution (spot) growth assay of wild type (WT), *mtm1*Δ, and *mtm1*Δ cells expressing either an empty vector (EV) or MOMTiP-4, assessed on CSM media supplemented with 2% glucose (fermentable) or 3% glycerol (respiratory) carbon source. Results are representative of three independent assays. (D) Mitochondrial iron content in wild-type (WT), *mtm1*Δ, and *mtm1*Δ cells complemented with MOMTiP-4, measured using a ferrozine-based colorimetric assay. Data are normalized to the wild-type (WT) control and presented as fold changes relative to WT (n=3) *p = 0.03. Error bars represent s.d. See also Figure S7.

**Figure 7.**
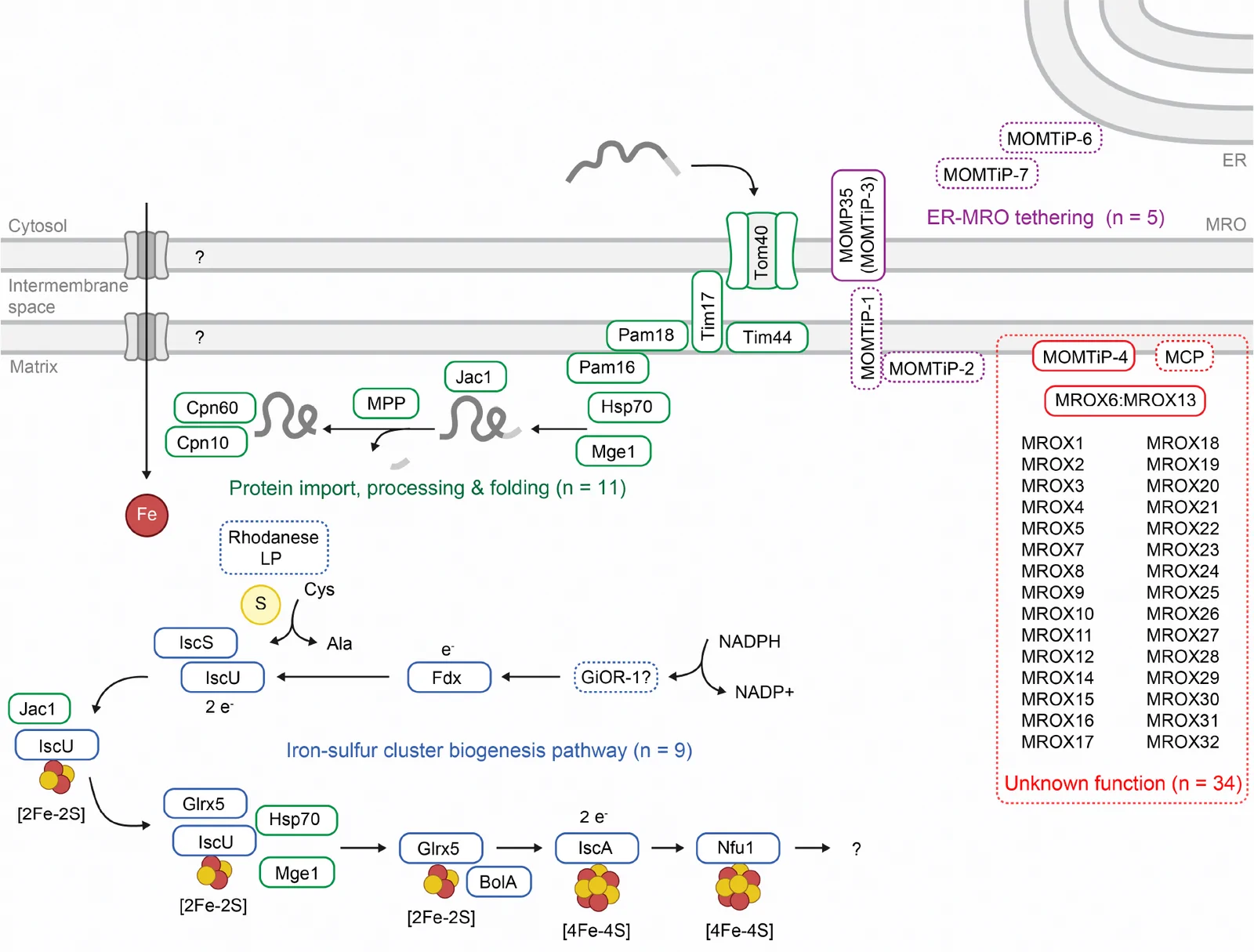
Pathway overview of GlMitoCarta proteome. Schematic overview of *Giardia* MRO proteins categorized into four main groups: (i) iron-sulfur cluster (ISC) assembly machinery (blue), including key components like cysteine desulfurase and scaffold proteins essential for ISC biogenesis; (ii) import and processing proteins (green), including translocases, chaperones, and peptidases that mediate protein import and maturation; (iii) ER-MRO tethering-like proteins (purple), which facilitate physical and functional interactions such as lipid exchange and signaling; and (iv) MRO proteins of unknown function (red), whose roles remain unclear. Additionally, proteins whose functions are currently speculative and lack definitive experimental evidence are illustrated with dotted outlines. This visual cue emphasizes the need for further validation to confirm their involvement in the proposed pathways or complexes within *Giardia’s* MRO system.

**KEY RESOURCES TABLE:** 

REAGENT or RESOURCE	SOURCE	IDENTIFIER
**Antibodies**		
Custom rabbit antibody raised against full length *Giardia* GiOR-1 (GL50803_0091252)	This study	N/A
Custom rabbit antibody raised against the N-terminal peptide of *Giardia* Tom40 (GL50803_0017161).	This study	N/A
Custom rabbit polyclonal antibody raised against full-length *Giardia* Tom40 (GL50803_0017161).	This study	N/A
Anti α-Tubulin Rabbit antibody	Cell Signaling	2125S
Anti HA-Tag Rabbit antibody	Cell Signaling	3724S
Anti HA-Tag Mouse antibody	Thermo Fisher	26183
Anti-PGK1 antibody	Thermo Fisher	PA5-28612
Anti MTCO1 antibody	Abcam	ab110270
Anti HA mouse antibody	Cell Signaling	2367S
Anti-Myc mouse antibody	Thermo Fisher Scientific	13-2500
Anti-rabbit Alexa-647	Thermo Fisher Scientific	A21244
Anti-Mouse Alexa 488	Thermo Fisher Scientific	A11001
DAPI (4′,6-diamidino-2-phenylindole)	Vector Laboratories	H-122-10
**Chemicals, peptides, and recombinant proteins**		
GiOR-1, recombinant protein, purified from *E.coli*	This study	N/A
6xHis-ULP1, recombinant protein, purified from *E.coli*	This study	N/A
*Giardia lamblia* Tom40 recombinant protein, purified from *E.coli*	This study	N/A
cOmplete^™^ Protease Inhibitor Cocktail	Sigma / Roche	11697498001
Proteinase K	Sigma / Roche	P5568-1ML
EveryBlot Blocking Buffer	Bio-Rad	12010020
Puromycin	InvivoGen	ant-pr-5b
Blasticidin	InvivoGen	ant-bl-5b
Deferoxamine mesylate	Sigma / Roche	D9533-1G
OptiPrep	Sigma / Roche	D1556
Benzonase	Sigma / Roche	E1014-25KU / E8263-5KU
Zymolyase	Zymo Research	E1004
Aprotinin	Sigma / Roche	A6103-25MG
Leupeptin	Sigma / Roche	11017128001
2X YT Broth, granulated	Research Products International	X15680-5000.0
Ni-NTA Agarose	Qiagen	30230
Phenylmethanesulfonyl fluoride solution, 0.1 M in ethanol (T)	Sigma-Aldrich	93482-50ML-F
Lysyl Endopeptidase, Mass Spectrometry Grade (Lys-C)	FUJIFILM Wako Pure Chemical Corporation	129-02541
Sequencing Grade Modified Trypsin	Promega	V5113
TRI Reagent Solution	Thermo Fisher Scientific	AM9738
UltraPure SDS Solution, 10%	Thermo Fisher Scientific	15553-035
Metronidazole	Sigma-Aldrich	M1547
Methanol	Honeywell	34966-4X4L
Pierce DTT (Dithiothreitol), No-Weigh Format	Thermo Scientific	A39255
Iodoacetamide	Sigma-Aldrich	A3221-10VL
Phosphoric Acid	LabChem	LC186401
Formic Acid	MilliporeSigma	5330020050
Empore C18 Extraction Disks	CSD Analytical	76333-132
Trifluoroacetic acid	Sigma-Aldrich	302031-10X1ML
1-Propanol	Sigma-Aldrich	279544-1L
PepSep C18 25cm × 75 μm, 1.5 μm	Bruker Daltonics	1893484
Acetonitrile	J.T.Baker	9829-03
**Critical commercial assays**		
Pierce^™^ BCA Protein Assay Kit	Thermo Fisher Scientific	23225
**Deposited data**		
*Giardia* MRO protein correlation profiling (MitoPCP) mass spectrometry proteomics dataset	This study	MSV000101929
*Giardia* metronidazole whole cell perturbation mass spectrometry proteomics dataset	This study	MSV000101929
*Giardia* Metronidazole, iron and O_2_ whole cell perturbation RNA-seq transcriptomics dataset	This study	GSE339042
*Giardia* IP- mass spectrometry proteomics dataset	This study	MSV000101929
*Giardia* MitoCarta confocal microscopy	This study	S-BIAD3383
**Experimental models: organisms/strains**		
*Giardia lamblia* strain WB clone C6	ATCC	50803
**Bacterial strains**		
NEB^®^ 5-alpha Competent *E.coli* (High Efficiency)	NEB	C2987H
NEB^®^ NiCo21(DE3) Competent *E.coli*	NEB	C2529H
BL21-CodonPlus (DE3)-RIPL Competent *E.coli*	Agilent	230280
**Yeast Strains**		
W303a (WT) (*MATa leu2-3 leu2-112 trp1-1 can1-100 ura3-1 ade2-1 his3-11*)	ATCC	208352
W303a *mtm1*Δ*::TRP1*	This study	N/A
W303a *aac2*Δ::*TRP1*	This study	N/A
W303a *leu5*Δ::*TRP1*	This study	N/A
W303a *mir1*Δ::*TRP1*	This study	N/A
W303a *sam5*Δ::*TRP1*	This study	N/A
W303a *ndt1*Δ::TRP1 *ndt2*Δ::*URA3*	This study	N/A
W303a *mrs3*Δ::TRP1 *mrs4*Δ::*URA3*	This study	N/A
W303a, *TEF1-MOMTiP-4-HA::LEU2*	This study	N/A
W303a, *mtm1*Δ*::TRP1 TEF1-MOMTiP-4-HA::LEU2*	This study	N/A
**Recombinant DNA**		
Plasmid: pKS-3xHA-PAC	Paredez lab (UW)	N/A
Plasmid: pKS-Epi_Puro_α-Tubulin_HaloTag-3xHA	This study	N/A
Plasmid: pKS-Epi_Puro_GS_GL50803_0014939-3xHA (MOMP35)	This study	N/A
Plasmid: pKS-Epi_BSR_GS_GL50803_0017161-2xMyc (Tom40)	This study	N/A
Plasmid: pKS-Epi_Puro_GS_GL50803_0091252-3xHA (GiOR-1)	This study	N/A
Plasmid: pKS-Epi_BSR_GS_GL50803_0015196-2xMyc (IscU)	This study	N/A
Plasmid: pKS-Epi_BSR_GS_GL50803_0010971-2xMyc (MOMTiP-2)	This study	N/A
Plasmid: pKS-Epi_Puro_GS_GL50803_008148-3xHA (MROX1)	This study	N/A
Plasmid: pKS-Epi_Puro_GS_GL50803_0017276-3xHA (MROX2)	This study	N/A
Plasmid: pKS-Epi_Puro_GS_GL50803_0010182-3xHA (MROX6)	This study	N/A
Plasmid: pKS-Epi_Puro_GS_GL50803_009296-3xHA (MOMTiP-4)	This study	N/A
Plasmid: pKS_Epi_Puro_GS_GL50803_0061303-3xHA (MROX7)	This study	N/A
Plasmid: pKS-Epi_Puro_GS_GL50803_005532-3xHA (MROX8)	This study	N/A
Plasmid: pKS-Epi_Puro_GS_GL50803_003202-3xHA (MROX9)	This study	N/A
Plasmid: pKS-Epi_Puro_GS_GL50803_0087492-3xHA (MROX10)	This study	N/A
Plasmid: pKS-Epi_BSR_GS_GL50803_0010452-2xMyc (Tim17)	This study	N/A
Plasmid: pKS-Epi_Puro_GS_GL50803_0015569-3xHA (MROX11)	This study	N/A
Plasmid: pKS-Epi_BSR_GS_GL50803_001376-2xMyc (Mge1)	This study	N/A
Plasmid: pKS-Epi_Puro_GS_GL50803_0027910-3xHA (RhodaneseLP)	This study	N/A
Plasmid: pKS-Epi_Puro_GS_GL50803_0011701-3xHA (MROX12)	This study	N/A
Plasmid: pKS-Epi_Puro_GS_GL50803_0017298-3xHA (MROX13)	This study	N/A
Plasmid: pKS-Epi_BSR_GS_GL50803_002013-2x-Myc (Glrx5)	This study	N/A
Plasmid: pKS-Epi_Puro_GS_GL50803_0029147-3xHA (MOMTiP-1)	This study	N/A
Plasmid: pKS-Epi_Puro_GS_GL50803_0029682-3xHA (MROX14)	This study	N/A
Plasmid: pKS-Epi_Puro_GS_GL50803_002848-3xHA (MROX15)	This study	N/A
Plasmid: pKS-Epi_Puro_GS_GL50803_0016581-3xHA (MROX16)	This study	N/A
Plasmid: pKS-Epi_Puro_GS_GL50803_0095290-3xHA (MROX17)	This study	N/A
Plasmid: pKS-Epi_Puro_GS_GL50803_008651-3xHA (MROX18)	This study	N/A
Plasmid: pKS-Epi_Puro_GS_GL50803_0029692-3xHA (MROX19)	This study	N/A
Plasmid: pKS-Epi_Puro_GS_GL50803_0040863-3xHA (MROX20)	This study	N/A
Plasmid: pKS-Epi_Puro_GS_GL50803_003491-3xHA (MROX3)	This study	N/A
Plasmid: pKS-Epi_Puro_GS_GL50803_0016677-3xHA (MROX21)	This study	N/A
Plasmid: pKS-Epi_Puro_GS_GL50803_0016424-3xHA (Disc AP)	This study	N/A
Plasmid: pKS-Epi_Puro_GS_GL50803_0017286-3xHA (MCP)	This study	N/A
Plasmid: pKS-Epi_Puro_GS_GL50803_005785-3xHA (MOMTiP-7)	This study	N/A
Plasmid: pKS-Epi_Puro_GS_GL50803_007188-3xHA (MROX4)	This study	N/A
Plasmid: pKS-Epi_Puro_GS_GL50803_0012229-3xHA (MROX5)	This study	N/A
Plasmid: pKS-Epi_Puro_GS_GL50803_005908-3xHA	This study	N/A
Plasmid: pKS-Epi_Puro_GS_GL50803_0010148-3xHA (MROX22)	This study	N/A
Plasmid: pKS-Epi_Puro_GS_GL50803_006519-3xHA (MROX23)	This study	N/A
Plasmid: pKS-Epi_Puro_GS_GL50803_008987-3xHA	This study	N/A
Plasmid: pKS-Epi_Puro_GS_GL50803_0014552-3xHA	This study	N/A
Plasmid: pKS-Epi_Puro_GS_GL50803_0015847-3xHA	This study	N/A
Plasmid: pKS-Epi_Puro_GS_GL50803_0014891-3xHA	This study	N/A
Plasmid: pKS-Epi_Puro_GS_GL50803_002226-3xHA	This study	N/A
Plasmid: TMIp025_pKS_Epi_Puro_GS_GL50803_0014627-3xHA	This study	N/A
Plasmid: TMIp026_pKS_Epi_Puro_GS_GL50803_0015367-3xHA (MROX27)	This study	N/A
Plasmid: TMIp027_pKS_Epi_Puro_GS_GL50803_009180-3xHA	This study	N/A
Plasmid: TMIp028_pKS_Epi_Puro_GS_GL50803_0015154-3xHA	This study	N/A
Plasmid: TMIp029_pKS_Epi_Puro_GS_GL50803_0024412-3xHA	This study	N/A
Plasmid: TMIp030_pKS_Epi_Puro_GS_GL50803_0031617-3xHA (MROX29)	This study	N/A
Plasmid: TMIp031_pKS_Epi_Puro_GS_GL50803_0014161-3xHA (MROX25)	This study	N/A
Plasmid: TMIp032_pKS_Epi_Puro_GS_GL50803_0012866-3xHA	This study	N/A
Plasmid: TMIp033_pKS_Epi_Puro_GS_GL50803_0022587-3xHA (MOMTiP-6)	This study	N/A
Plasmid: TMIp034_pKS_Epi_Puro_GS_GL50803_007135-3xHA (MROX28)	This study	N/A
Plasmid: TMIp035_pKS_Epi_Puro_GS_GL50803_008657-3xHA (MROX24)	This study	N/A
Plasmid: TMIp036_pKS_Epi_Puro_GS_GL50803_0016640-3xHA	This study	N/A
Plasmid: TMIp037_pKS_Epi_Puro_GS_GL50803_008102-3xHA (MROX26)	This study	N/A
Plasmid: TMIp038_pKS_Epi_Puro_GS_GL50803_003117-3xHA	This study	N/A
Plasmid: TMIp039_pKS_Epi_Puro_GS_GL50803_0011196-3xHA	This study	N/A
Plasmid: TMIp040_pKS_Epi_Puro_GS_GL50803_0014553-3xHA (MROX31)	This study	N/A
Plasmid: TMIp041_pKS_Epi_Puro_GS_GL50803_0012213-3xHA	This study	N/A
Plasmid: TMIp042_pKS_Epi_Puro_GS_GL50803_005859-3xHA (MROX32)	This study	N/A
Plasmid: TMIp043_pKS_Epi_Puro_GS_GL50803_003307-3xHA (MROX30)	This study	N/A
Plasmid: 6xHis-SUMO-GL50803_0091252 (GiOR-1)	This study	N/A
Plasmid: 6xHis-GL50803_0017161 (Tom40)-pET28a	This study	N/A
Plasmid: pRS405-TEF1-*LEU2*	Addgene	15968
Plasmid: pTEF1-MOMTiP-4-HA-*LEU2*	This study	N/A
**Software and algorithms**		
Excel for Mac/Windows	Microsoft	Version 16.102.3
PyMOL	Schrödinger, LLC	Version 2.5.4
Prism	Dotmatics	Version 10.6.1
Snapgene	Dotmatics	Version 8.1.1
FIJI (ImageJ)	National Institutes of Health	ImageJ 1.54p
Adobe Illustrator	Adobe	Version 29.3.1
**Other**		
Pre-Separation Filters (30 μm)	Miltenyi	130-041-407
LS Columns	Miltenyi	130-042-401
QuadroMACS^™^, magnetic separator and multi stand	Miltenyi	130-091-051
μMACS^™^ HA Isolation Kit	Miltenyi	130-091-122
Vinyl Anaerobic Chamber	Coy Lab Products	Type C
Direct-zol RNA Miniprep Plus	Zymo Research	R2071
NAb^™^ Protein A Plus Spin Column kit	Thermo Fisher Scientific	89960
Clarity Western ECL Substrate	Bio-Rad	1705061
Q5 Hotstart Master Mix 2x	New England Biolabs	M0494S
NEBuilder HiFi DNA Assembly Master Mix	New England Biolabs	E2621
Novex^™^ WedgeWell^™^, 4–20% Tris-Gly protein gels	Thermo Fisher Scientific	XP04200BOX
S-Trap Micro Column	PROtiFI	C02-micro-40
Sep-Pak tC18 96-well Plate, 40 mg Sorbent per Well	Waters	186002320
Aurora Elite 15×75 CSI C18 UHPLC column	IonOpticks	AUR4-15075C18-CSI
PureLink^™^ HiPure Plasmid Midiprep Kit	Thermo Fisher Scientific	K210005
Gene Pulser/MicroPulser Electroporation Cuvettes (0.4 cm)	Bio-Rad	1652088
Ultracentrifuge tube (14 × 89 mm, Polypropylene)	Seton Scientific	7030
Ultracentrifuge tubes (16 × 102 mm, Ultra-Clear)	Beckman Coulter	344061
Ultracentrifuge tubes (25 × 89 mm, Ultra-Clear)	Beckman Coulter	C14292

## Highlights

- Proteomics and microscopy yield the *Giardia* MitoCarta with 59 proteins
- 25 MRO proteins are of known function: Import, ISC biogenesis, ER-MRO tethering
- Metronidazole treatment leads to modest elevation of MRO protein levels
- MOMTiP-4 functionally complements the yeast solute carrier Mtm1
