# Supplementary material for: A high-confidence inventory of the proteins in the mitochondrion-related organelle of Giardia
Source: Curr Biol. Author manuscript; Available in PMC 2026 Oct 8. (PMC13643923; doi:10.1016/j.cub.2026.07.074)
Supplement: Supplementary file 1 [file NIHMS2204267-supplement-8.pdf]

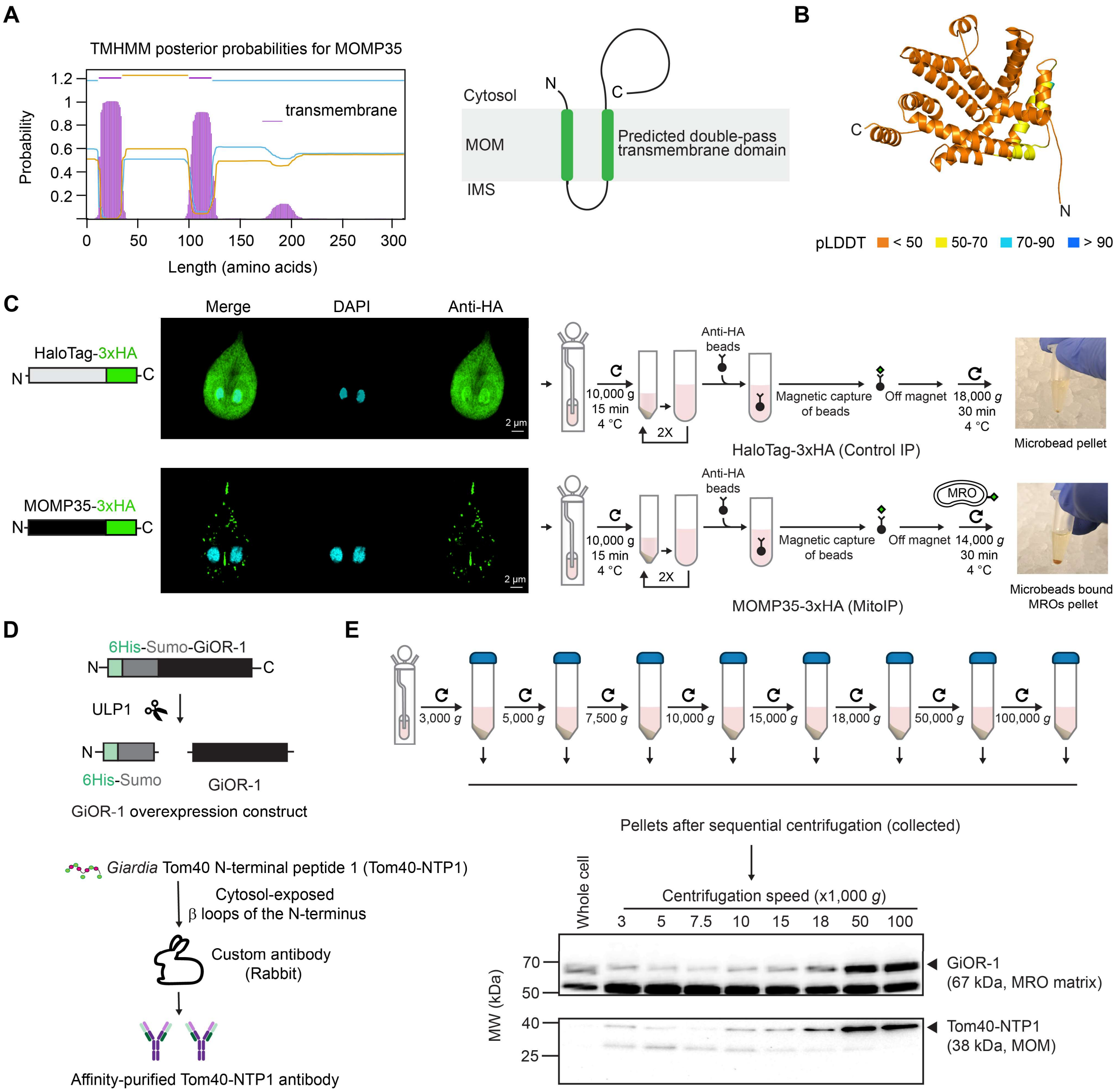


**Figure S1. Construct design, validation, and MitoIP optimization, related to Figure 1.**
(A) MOMP35 transmembrane domain and topology predicted by the Transmembrane Helices Hidden Markov Model (TMHMM).
(B) Predicted 3D structure of MOMP35 showing helical features, including the two α-helices located at the N-terminal end, spanning the MRO outer membrane (MOM), while the C-terminus faced towards the cytosol. The 3D structure is colored by predicted local distance difference test (pLDDT).
(C) Construct design and confocal imaging of *Giardia* trophozoites transfected with either HaloTag-3xHA (cytosolic) or MOMP35-3xHA (MRO), anti-HA construct stained in green (Alexa Fluor 488), while nuclear DNA (DAPI) stained as cyan. Right panel shows scheme of affinity purification from *Giardia* transfected with either MOMP35-3xHA or HaloTag-3xHA using anti-HA magnetic beads. Only the MOMP35-3xHA parasite resulted in a solid microbead pellet with extracted MROs.

(D) Schematic depicting (i) the design of the recombinant 6xHis-SUMO-GiOR-1 fusion protein construct and the cleavage mechanism of the His-SUMO tag by ULP1 protease (upper panel), (ii) peptide-based generation of the anti-Tom40 antibody, targeting cytosol-exposed beta loops at the N-terminus (NTP1) (lower panel).
(E) Screening of rabbit anti-Tom40-NTP1 (MRO outer membrane) and anti-GiOR-1 (MRO matrix) antibodies on sequential differential centrifugation fractions from *Giardia* trophozoites' soluble lysates.

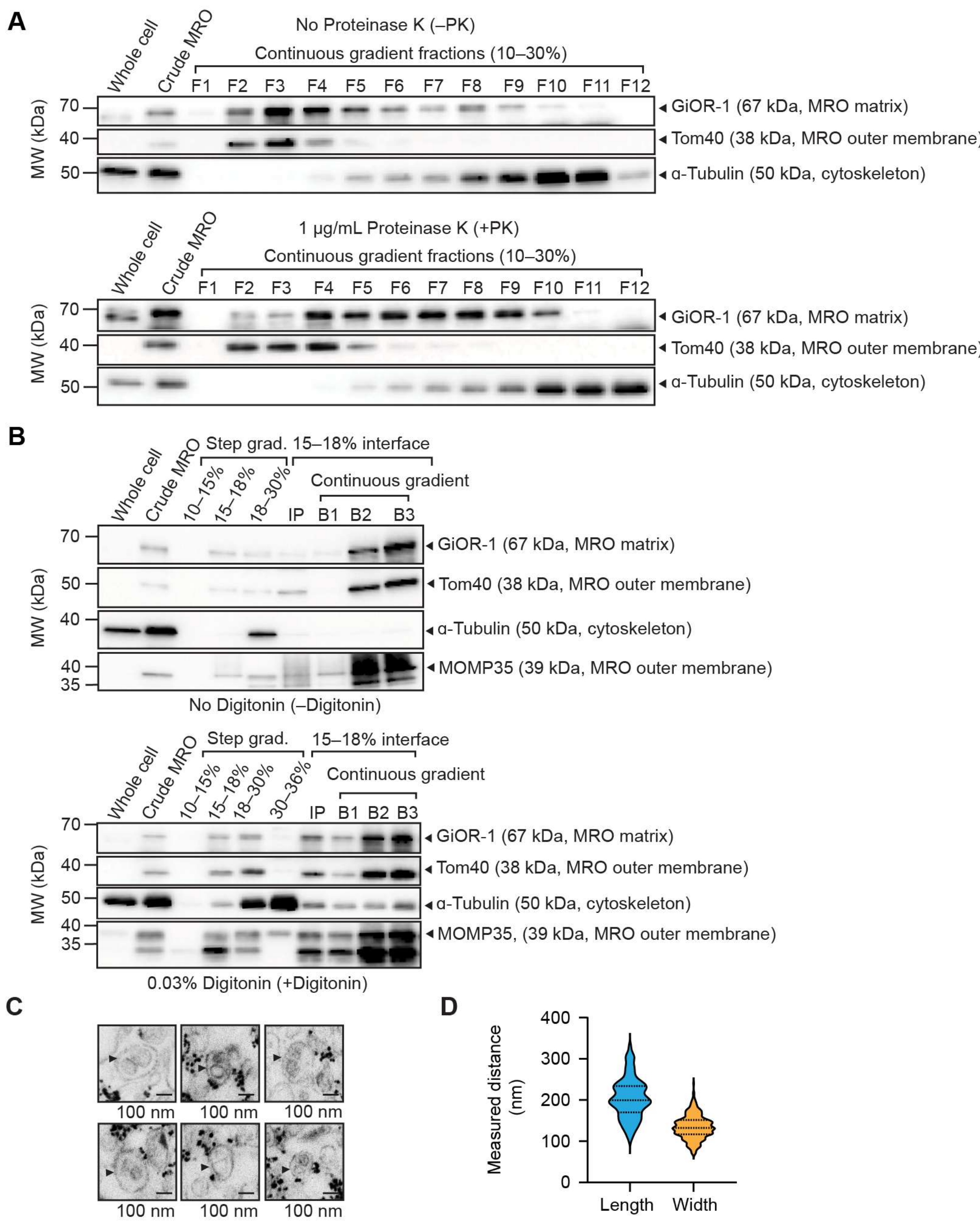


**Figure S2. Immunoblot and TEM analysis of isolated MRO fractions: Experimental validation and quality assessment, related to Figure 2.**

(A) Western blot analysis of fractions collected during wild-type MRO isolation (Workflow 1), untreated (top panels) or treated (bottom panels) with proteinase K. GiOR-1 (matrix), anti-Tom40 (outer membrane), and anti-HA (MOMP35-3xHA construct, outer membrane) antibodies served as MRO markers, while anti-α-tubulin served as a control to detect cytosol/cytoskeletal contamination. There was a shift in GiOR-1 intensity to higher density fractions and a decrease in tubulin intensity after proteinase K treatment.

(B) Immunoblots of fractions collected during MitoIP and subsequent continuous density gradient fractionation from MOMP35-3xHA-transfected parasites (Workflow 2). Parasite lysates were either untreated (top panels) or treated with 0.03% digitonin (bottom panels). With digitonin treatment, there was an increase in tubulin band intensity.

(C) High-magnification transmission electron microscopy (TEM) micrographs from different steps of Workflow 2 (MitoIP and continuous density gradient purified fractions B2 and B3), revealing well-defined segmentation and internal compartmentalization of MROs.
(D) Quantitative statistics of purified MROs (Workflow 2, continuous density band B3), including measurements of length and width (n = 345).

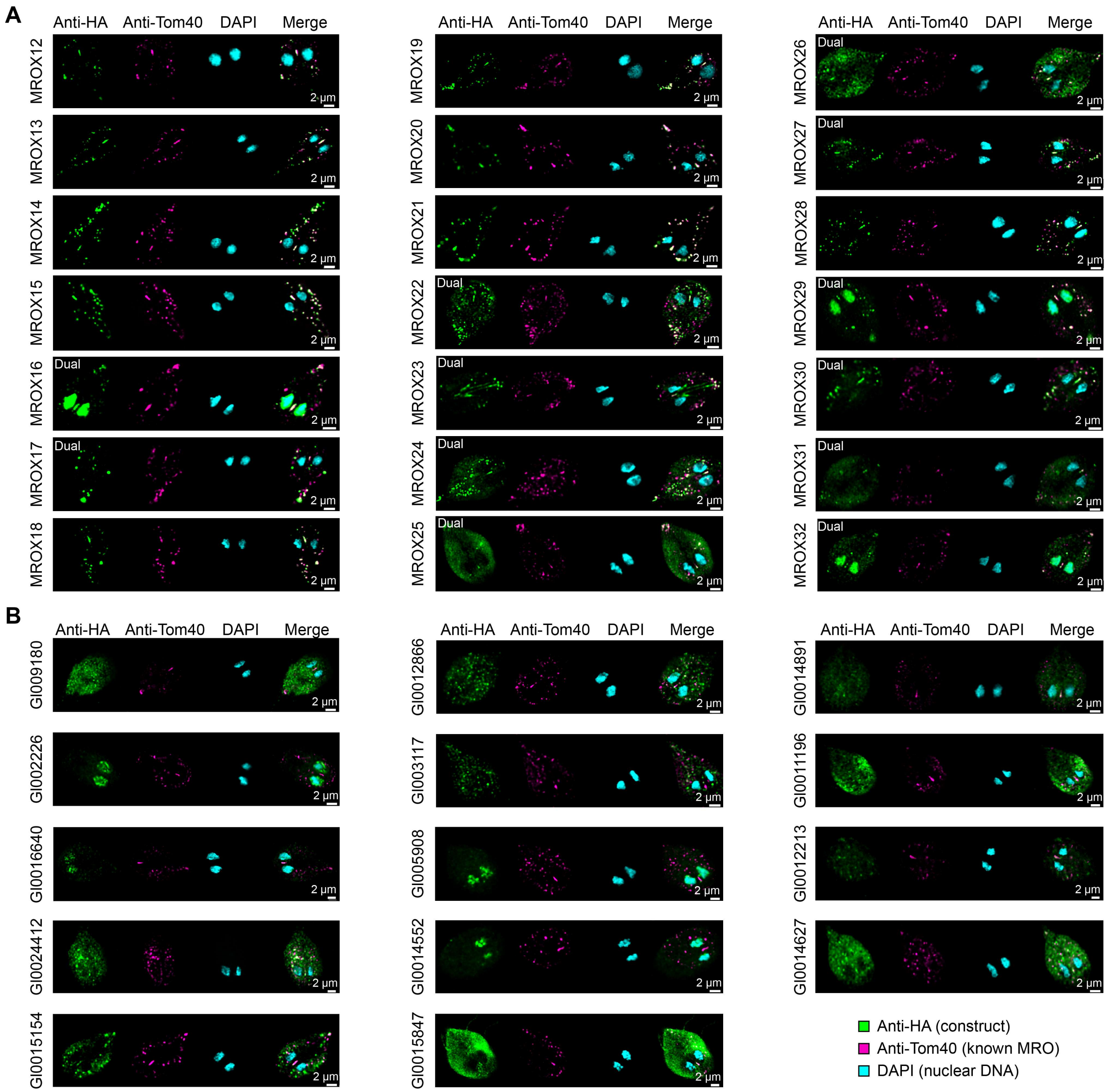


**Figure S3. Validation of MitoViaPCP identified proteins by confocal microscopy, related to Figure 3**.
(A) Confocal immunofluorescence microscopy of MitoViaPCP proteins (PCP score ≥ 0.84) categorized as MRO localized. Proteins were expressed as 3xHA-tagged constructs and showed colocalization with the MRO marker Tom40. Anti-HA antibodies (green), anti-Tom40 antibodies (magenta) and nuclear DNA was visualized by DAPI staining (cyan). Some of the candidates are localized to both MRO and other subcellular structures labeled dual.
(B) Confocal immunofluorescence microscopy of MitoViaPCP candidates (PCP score ≥ 0.84) categorized as non-MRO due to lack of sufficient colocalization with Tom40. Anti-HA antibodies (green), anti-Tom40 antibodies (magenta) and nuclear DNA was visualized by DAPI staining (cyan).

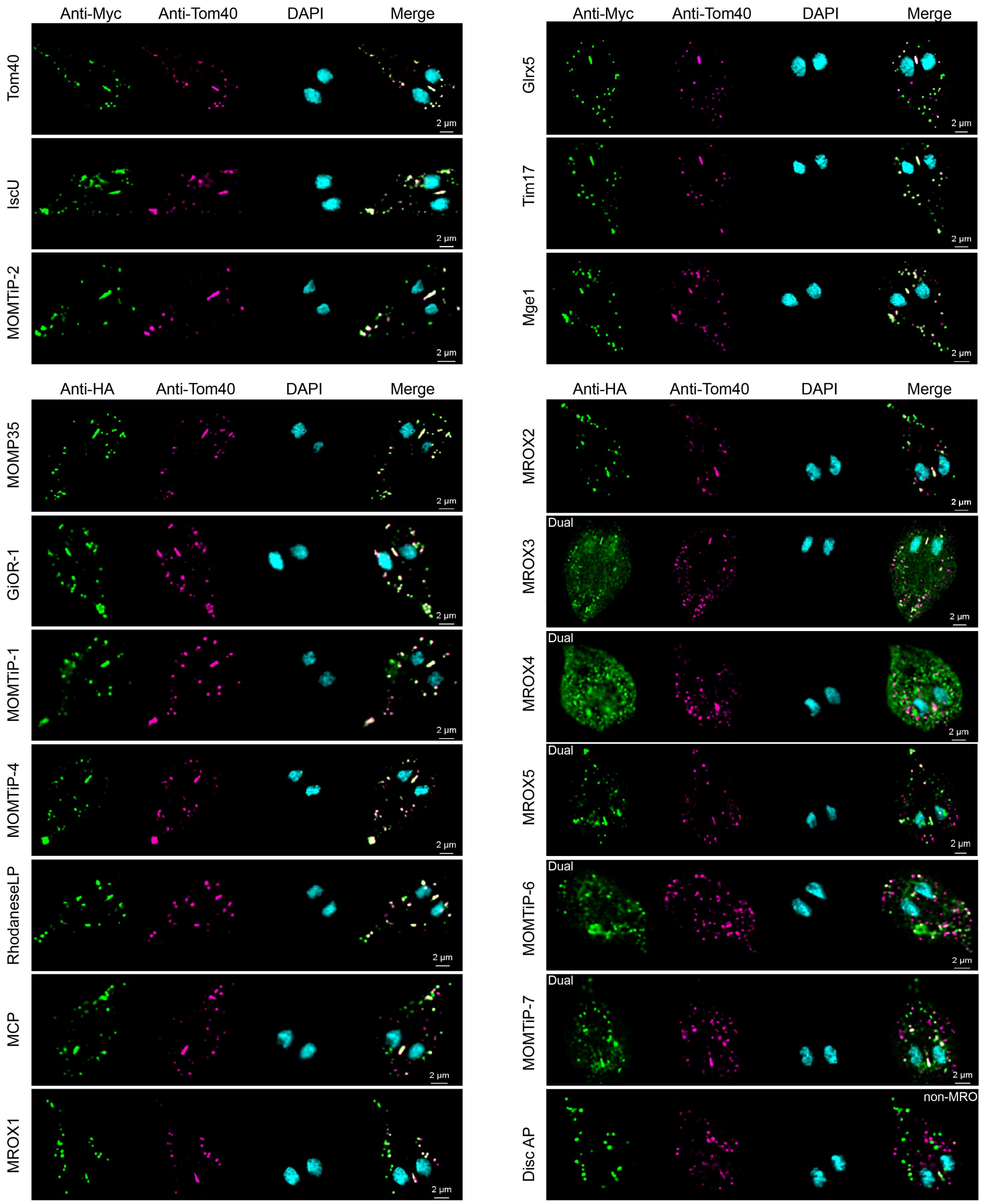


**Figure S4. Validation of known MRO proteins (gold+) by confocal microscopy, related to Figure 3**. Confocal immunofluorescence microscopy of *Giardia* known MRO proteins (gold+) expressed as either 2xMyc or 3xHA-tagged constructs with MRO marker Tom40, as indicated. Anti-HA or anti-Myc antibodies (green), anti-Tom40 antibodies (magenta) and nuclear DNA was visualized by DAPI staining (cyan). Five gold+ proteins, which scored below the PCP threshold, showed localization to MRO and other subcellular structures (marked as dual).

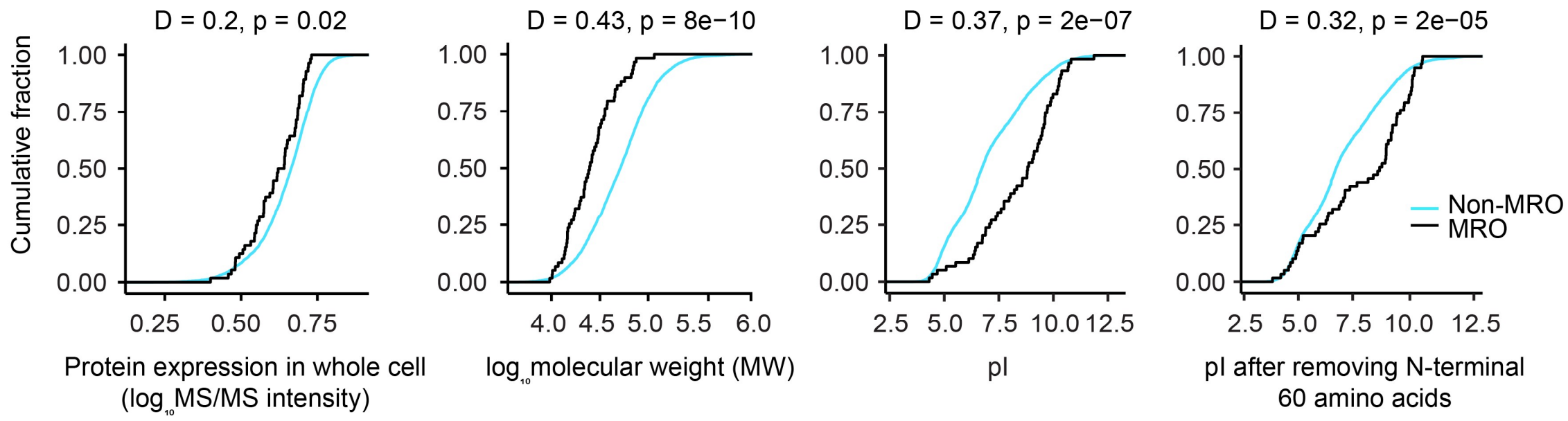


**Figure S5. Characteristics of the GlMitoCarta, related to Figure 4.** Cumulative distribution function (CDF) plots for *Giardia* MitoCarta proteins (black, MRO) compared to non-MRO proteins (cyan). pI indicates isoelectric point (BioPerl pICalculator).

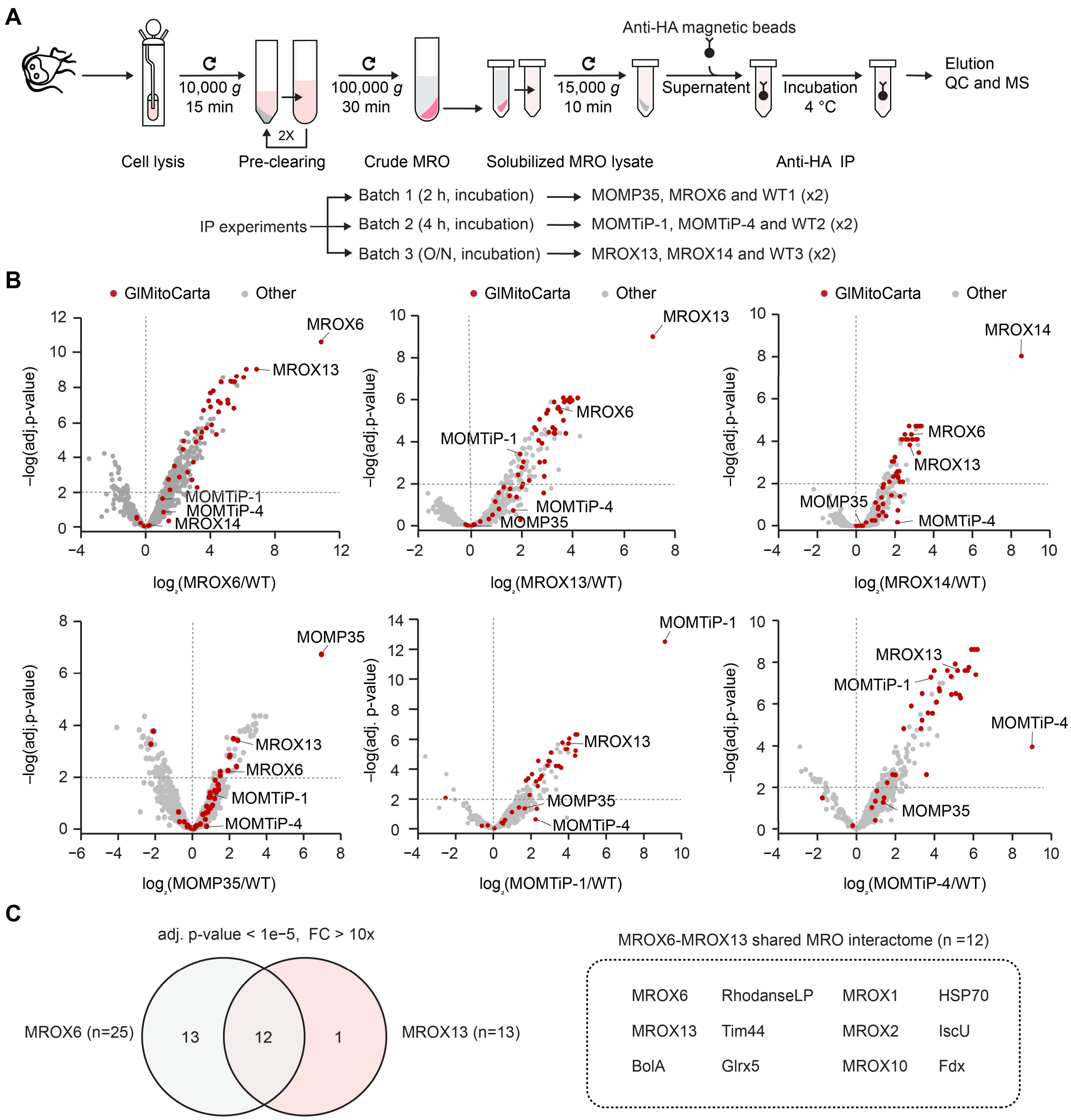


**Figure S6. Functional analysis of GlMitoCarta proteins of unknown function, related to Figure 5.** (A) Workflow for crude MRO isolation from wild-type (WT) and transfected parasites (expressing 3xHA-tagged bait protein) using differential centrifugation, followed by immunoprecipitation with anti-HA magnetic beads. IP experiments were performed in biological duplicates across three independent batches, each paired with their WT MRO lysate IP, as control.

(B) Volcano plots show $log_2$(target/WT) versus significance for proteins detected in each of six IP-MS (target displayed on X-axis). GlMitoCarta proteins are shown in red and non-MRO proteins in grey. The dashed line indicates adj. p-value < 0.01.

(C)Venn diagram illustrates the MROX6 and MROX13 interactomes. High-confidence threshold (fold change >10× and adjusted p-value < $1 e^{-5}$) identifies 25 MRO proteins in MROX6 and 13 in MROX13, while 12 are shared between the two IP-MS analyses (highlighted in the right panel).

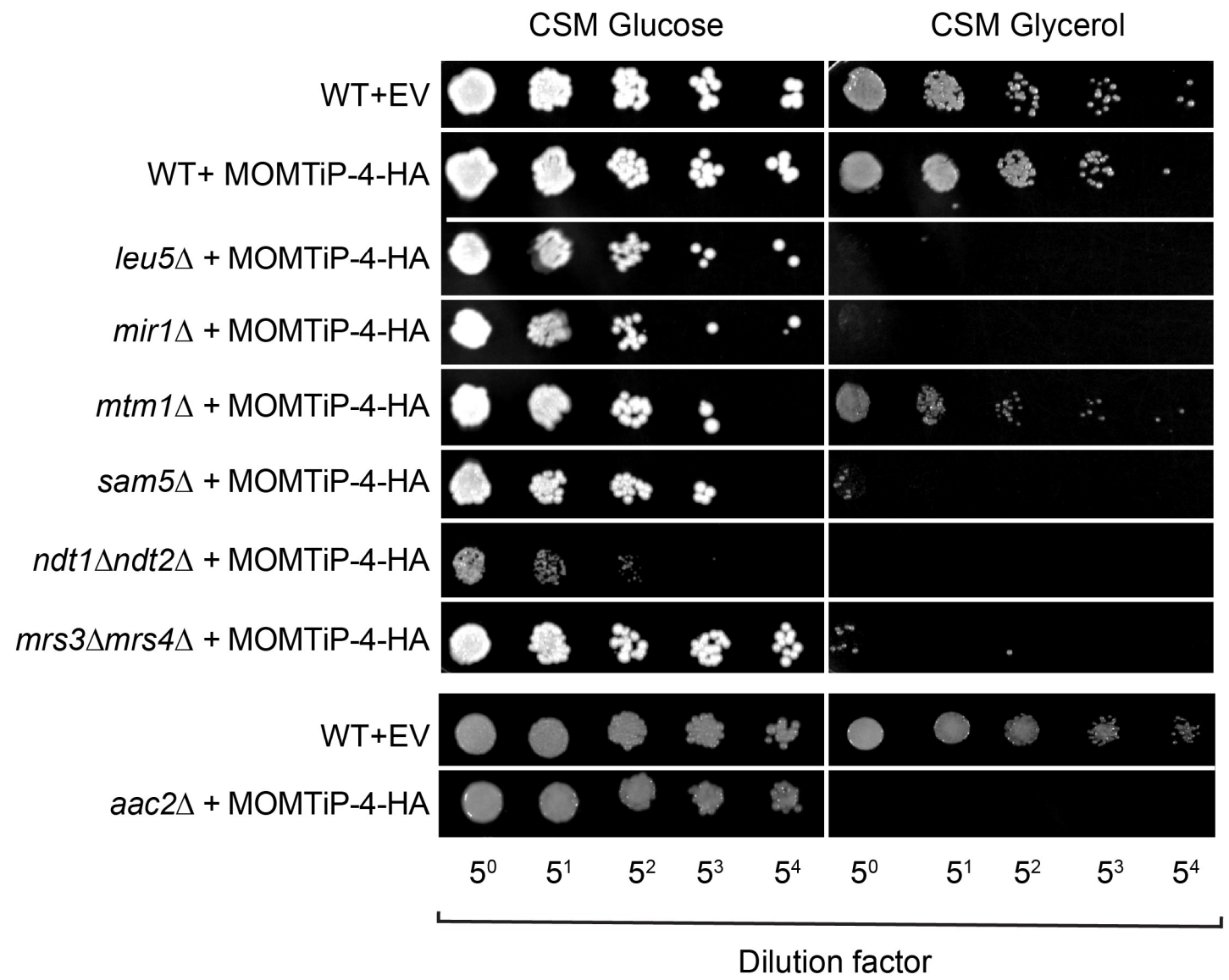


**Figure S7. Functional complementation of MOMTiP-4 in *Saccharomyces cerevisiae* SLC25A mitochondrial carrier family mutants, related to Figure 6.** Serial dilution (spot) growth assays were performed using wild-type (WT) cells, single knockout strains (*aac2*Δ, *leu5*Δ, *mir1*Δ, *mtm1*Δ, *sam5*Δ), and double knockout (*ndt1*Δ *ndt2*Δ, *mrs3*Δ *mrs4*Δ) backgrounds of the yeast SLC25A mitochondrial carrier family. These strains were transformed with either an empty vector (EV) or MOMTiP-4 as indicated. Growth was assessed on CSM media supplemented with either 2% glucose (fermentable carbon source) or 3% glycerol (non-fermentable respiratory carbon source) to evaluate functional complementation of growth defects.
